# Chromatin factor YY1 controls fetal hematopoietic stem cell migration and engraftment in mice

**DOI:** 10.1172/JCI188140

**Published:** 2025-07-29

**Authors:** Sahitya Saka, Zhanping Lu, Yinghua Wang, Peng Liu, Deependra K. Singh, Junki P. Lee, Carmen G. Palii, Tyler R. Alvarez, Anna L.F.V. Assumpção, Xiaona You, Jing Zhang, Marjorie Brand, Michael L. Atchison, Xuan Pan

**Affiliations:** 1 Department of Medical Sciences, School of Veterinary Medicine;; 2 Carbone Cancer Center;; 3 Department of Biostatistics and Medical Informatics, School of Medicine and Public Health;; 4 Department of Cell and Regenerative Biology, School of Medicine and Public Health;; 5 Wisconsin Blood Cancer Research Institute; and; 6 McArdle Laboratory for Cancer Research, School of Medicine and Public Health, University of Wisconsin–Madison, Madison, Wisconsin, USA.; 7 Department of Biomedical Sciences, School of Veterinary Medicine, University of Pennsylvania, Philadelphia, Pennsylvania, USA.

**Keywords:** Development, Hematology, Embryonic development, Hematopoietic stem cells

## Abstract

The fetal liver is the primary site of hematopoietic stem cell (HSC) generation during embryonic development. However, the molecular mechanisms governing the transition of hematopoiesis from the fetal liver to the BM remain incompletely understood. Here, we identify the mammalian Polycomb Group protein Yin Yang 1 (YY1) as a key regulator of this developmental transition. Conditional deletion of *Yy1* in the hematopoietic system during fetal development results in neonatal lethality and depletion of the fetal HSC pool. YY1-deficient fetal HSCs exhibit impaired migration and fail to engraft in the adult BM, thereby losing their ability to reconstitute hematopoiesis. Transcriptomic analysis reveals that *Yy1* KO disrupts genetic networks controlling cell motility and adhesion in fetal hematopoietic stem and progenitor cells (HSPCs). Notably, YY1 does not directly bind the promoters of most dysregulated genes. Instead, it modulates chromatin accessibility at regulatory loci, orchestrating broader epigenetic programs essential for HSPC migration and adhesion. Together, these findings establish YY1 as a critical epigenetic regulator of fetal HSC function and provide a mechanistic framework to further decipher how temporal epigenomic configurations determine HSC fetal-to-adult transition during development.

## Abstract 2

Chromatin factor YY1 is essential for fetal-to-adult transition in hematopoiesis and controls fetal HSC engraftment to adult bone marrow via distinct direct and indirect mechanisms.

## Graphical abstract

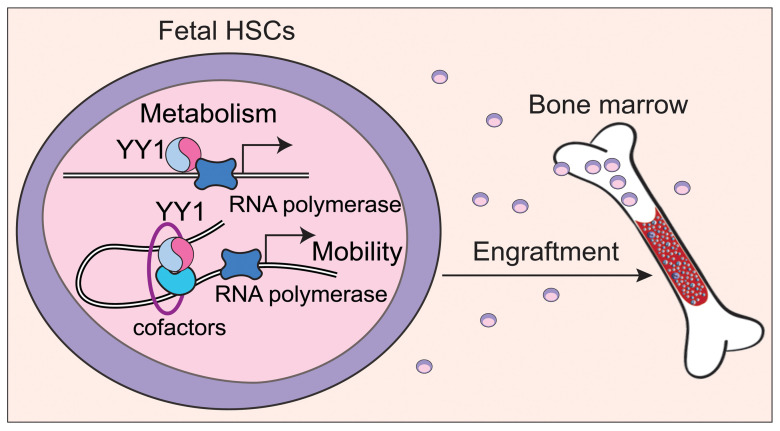

## Introduction

Hematopoietic stem cells (HSCs) first emerge in the aorta-gonad-mesonephro (AGM) region of the mouse embryo around E10.5 (1–3). These AGM-derived HSCs subsequently migrate to the fetal liver (FL), where they undergo rapid expansion between E12 and E16 (4). Around E16.5–17.5, fetal HSCs begin to migrate to the BM, which thereafter becomes the primary site of hematopoiesis throughout adulthood (5). The successful migration and anchorage of fetal HSCs within the BM niche are mediated by multiple receptor-ligand interactions, including stem cell factor (SCF)/c-Kit signaling, which is essential during fetal development (6–10). In addition, the fetal-to-adult transition of hematopoiesis is accompanied by profound changes in 3D genome organization, particularly in long-range enhancer-promoter interactions within topologically associated domains (11, 12). These developmental stage–specific chromatin interactions, regulated largely by transcription factors, drive gene expression programs that distinguish fetal from adult HSCs (11–16). However, the precise mechanisms by which transcription factor–mediated 3D genome organization regulates fetal hematopoiesis remain largely unknown.

Yin Yang 1 (YY1) is a ubiquitously expressed zinc finger transcription factor and mammalian Polycomb Group (PcG) protein involved in diverse biological processes, including early embryo development, X chromosome inactivation, DNA repair, cell cycle progression, apoptosis, and hematopoiesis (17–19). Germline deletion of YY1 leads to peri-implantation lethality, underscoring its essential role in embryogenesis (20). Our previous study demonstrated that YY1 is a critical regulator of adult HSC self-renewal and quiescence, and it directly regulates SCF/c-Kit signaling in adult HSCs (21). Beyond its role in transcriptional regulation, YY1 also plays essential roles in shaping higher-order chromatin architecture. It colocalizes with chromatin structural regulators such as CCCTC-binding factor (CTCF) and cohesin to regulate long-range chromatin interactions, bringing enhancers and promoters into spatial proximity (22–26). In adult hematopoietic stem progenitor cells (HSPCs), YY1 colocalizes with cohesin throughout the genome and facilitates developmentally regulated changes in chromatin architecture (27). Although FL HSCs undergo a developmental transition to adult BM HSCs around 3–4 weeks after birth (28), their physiological and functional characteristics remain distinct (29–32). Despite YY1’s established role in adult hematopoiesis and chromatin organization, its function during fetal HSC development remains undefined. A central unanswered question is how a ubiquitously expressed transcription factor like YY1 mediates developmental stage–specific regulatory programs in hematopoiesis.

Herein, we assessed YY1 functions in fetal hematopoiesis by utilizing the *Yy1^fl/fl^ Vav-Cre* mouse model. Our results show that deletion of YY1 in the hematopoietic system during fetal development leads to neonatal lethality and significantly decreased HSC numbers. YY1-deficient fetal HSCs have reduced capacity of migration and fail to engraft the adult BM. YY1 deficiency disrupts the genetic network governing cell motility, adhesion, and metabolism and impairs SCF/c-Kit signaling in fetal HSPCs. Mechanistically, YY1 does not bind directly to the promoters of the majority of deregulated genes and regulates fetal HSPC migration and engraftment largely by indirect mechanisms. YY1 functions in concert with various transcriptional factors and chromatin regulators across the genome to modulate both global and locus-specific chromatin accessibility, particularly at gene loci that are essential for cell adhesion and mobility. Importantly, fetal HSCs lacking the YY1 REPO domain (*Yy1^–/∆REPO^*), which is required for PcG function but retains transcriptional activation and repression capacities, fail to reconstitute blood. This indicates that the YY1 REPO domain is critical for fetal HSC long-term self-renewal. In conclusion, YY1 supports fetal HSC function through a combination of direct transcriptional control and indirect epigenetic mechanisms. We uncover important epigenetic mechanisms by which a ubiquitously expressed transcription factor mediates developmental stage–specific requirements in hematopoiesis.

## Results

### Conditional YY1 deletion in the mouse fetal hematopoietic system leads to neonatal death.

**Figure 1:**
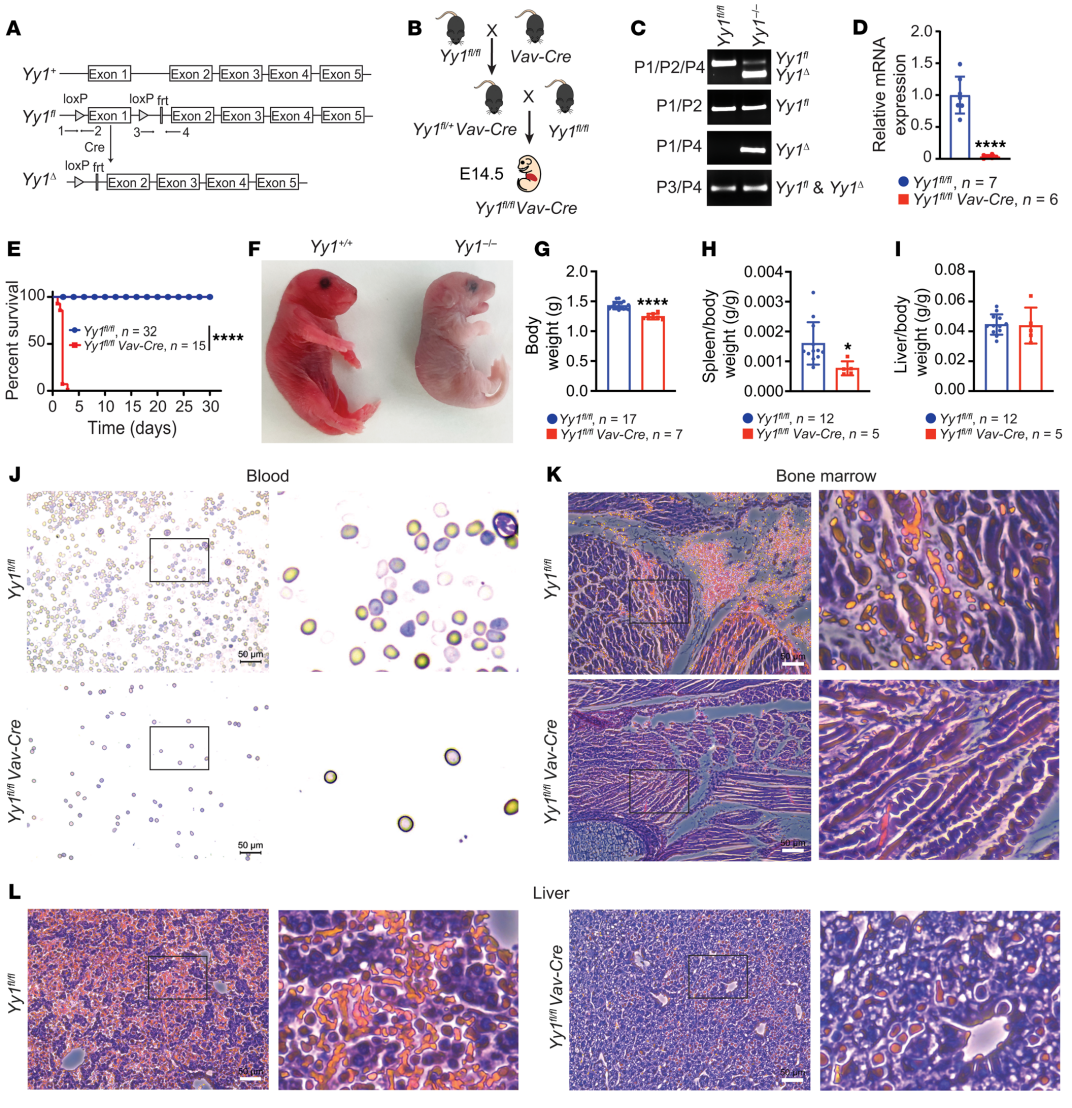
*Yy1* deletion during embryonic hematopoiesis leads to neonatal death in mice. (**A**) Schematic illustration of the *Yy1* locus. The conditional *Yy1* allele (*Yy1^fl^*) was constructed by inserting a pair of loxP sites flanking exon 1 at the promoter region, which is excised in the presence of Cre recombinase expression. (**B**) Illustration of breeding strategy to generate *Yy1^fl/fl^ Vav-Cre* fetuses. (**C**) PCR to detect YY1 deletion efficiency in total FL cells. Primers 1 and 2 detect *Yy1^fl^*. Primers 1 and 4 detect *Yy1^Δ^*. Primers 3 and 4 detect both *Yy1^fl^* and *Yy1^Δ^*. Mixed primers 1, 2, and 4 showed the relative primer efficiency. (**D**) The mRNA expression of *Yy1* gene in the LSK cells of E14.5 *Yy1^fl/fl^ Vav-Cre* fetus as compared with *Yy1^fl/fl^* littermates. (**E**) Kaplan-Meier survival curve of *Yy1^fl/fl^* and *Yy1^fl/fl^ Vav-Cre* mice. (**F**) A representative photo of *Yy1^fl/fl^ Vav-Cre* and *Yy1^fl/fl^* neonates around 24 hours after birth. (**G**) Body weight, (**H**) spleen/body weight, and (**I**) liver/body ratios of *Yy1^fl/fl^ Vav-Cre* and *Yy1^fl/fl^* mice. (**J**) Images of blood smears and histopathology of BM (**K**) and liver (**L**) of *Yy1^fl/fl^ Vav-Cre* and *Yy1^fl/fl^* mice. (**J**–**L**) Scale bar: 50 μm. Number of mice = *n*; graphs show mean ± SD, *P < 0.05, ****P < 0.0001, by unpaired 2-tailed Student’s *t* test (**D** and **G**–**I**) and simple survival curve analysis (Kaplan-Meier) (**E**).

Given that YY1 germline deletion causes peri-implantation lethality (20), we utilized a conditional *Yy1*-KO allele (*Yy1^fl^*) with loxP sites flanking the *Yy1* promoter region and exon 1 (17) to investigate the impact of YY1 loss of function on fetal hematopoiesis (Figure 1A). *Yy1^fl/fl^* mice were crossed to *Vav-Cre* mice to generate heterozygous *Yy1^fl/+^* *Vav-Cre* mice. The *Vav* promoter drives Cre recombinase expression specifically in the hematopoietic system starting at E11.5 of fetal development (33–35). *Yy1^fl/+^* *Vav-Cre* mice were subsequently bred with *Yy1^fl/fl^* mice to generate homozygous *Yy1^fl/fl^ Vav-Cre* (*Yy1^–/–^*) mice (Figure 1B). At E14.5 of fetal development, *Yy1* gene alleles were deleted in FL cells (Figure 1C). Compared with *Yy1^fl/fl^* (*Yy1^+/+^*) littermate controls, *Yy1^fl/fl^ Vav-Cre* mice exhibited a 90% reduction of YY1 transcript expression in FL Lin^–^Sca1^+^c-Kit^+^ (LSK) cells (Figure 1D). Although *Yy1^fl/fl^ Vav-Cre* fetuses were viable at E14.5 and E19.5 of fetal development, the ratios of surviving embryos were less than the predicted Mendelian frequency (<25%). In addition, no *Yy1^fl/fl^ Vav-Cre* mice were viable at the weaning stage (Table 1). Among 162 pups resulting from breeding *Yy1^fl/fl^* to *Yy1^fl/+^* *Vav-Cre*, only 15 pups were *Yy1^fl/fl^ Vav-Cre*, indicating only around 37% (15/40) of YY1-deficient fetuses survive until birth. All 15 pups died within 72 hours after birth (Figure 1E). Compared with *Yy1^fl/fl^* littermate controls, *Yy1^fl/fl^ Vav-Cre* pups were small and pale (Figure 1F) with significantly reduced body weight (Figure 1G) and spleen/body weight ratio (Figure 1H) and normal liver/body weight ratio (Figure 1I). *Yy1^fl/fl^ Vav-Cre* pups had reduced red blood cells and white blood cells in peripheral blood (Figure 1J), BM (Figure 1K), and liver (Figure 1L). Our results showed that YY1 deletion in fetal hematopoiesis leads to pancytopenia and neonatal death.

### YY1 is essential for maintaining the HSC pool in fetal hematopoiesis.

**Figure 2:**
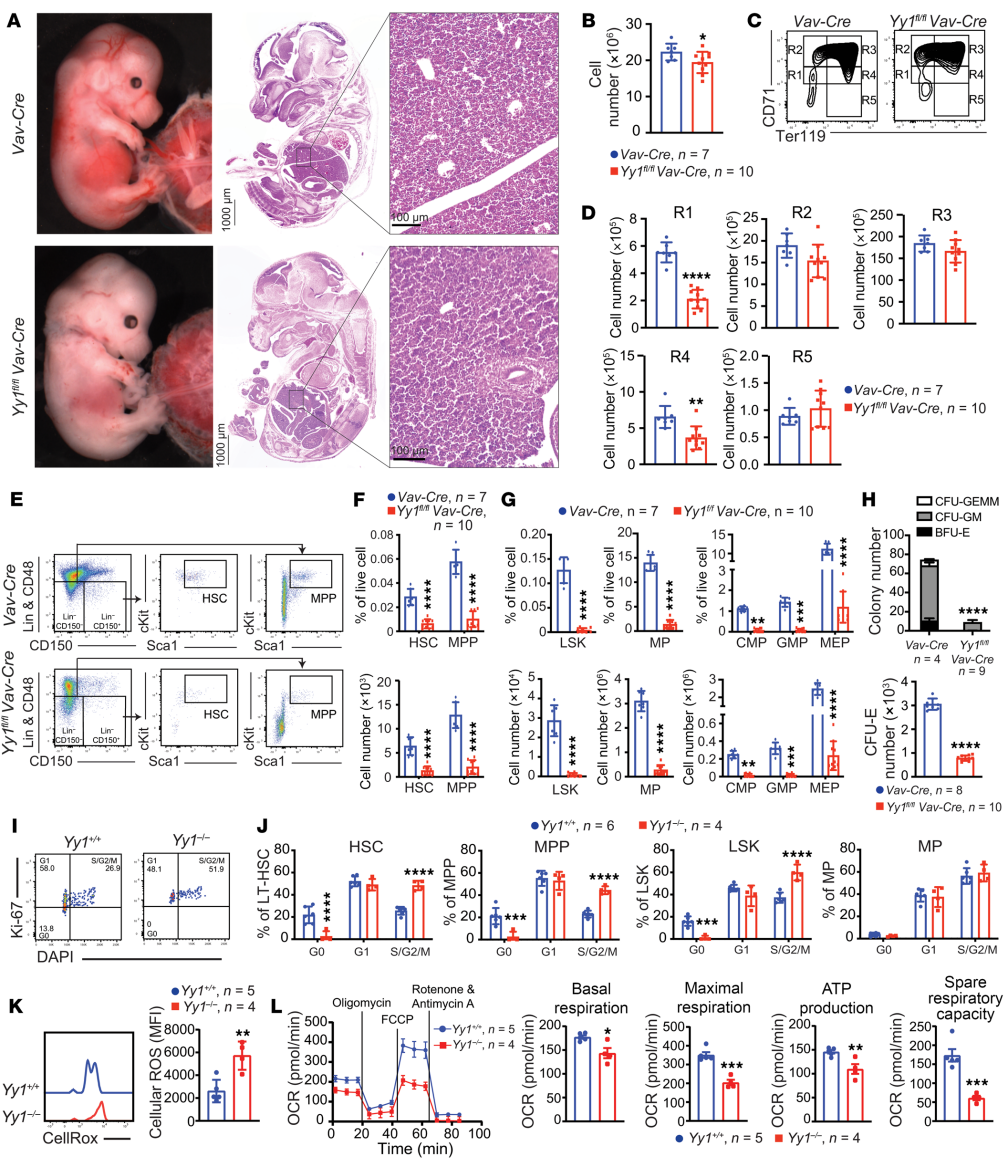
Loss of *Yy1* leads to reduction of HSC and progenitor populations in FL. All *Yy1^fl/fl^ Vav-Cre* and *Vav-Cre* fetuses were evaluated at E14.5 of fetal development. (**A**) Representative gross pictures; histopathology pictures of fetuses and FLs. (**B**) Total FL cell numbers. (**C**) Red blood cell flow analysis and (**D**) quantification. (**E**) Representative flow gating strategy for HSC (Lin^–^CD41^–^CD48^–^Mac1^+^CD150^+^cKit^+^Sca1^+^) and MPP (Lin^–^CD41^–^CD48^–^Mac1^+^CD150^–^cKit^+^Sca1^+^). (**F**) Quantification of percentage and absolute cell numbers of HSC and MPP. (**G**) Quantification of LSK (Lin^–^cKit^+^Sca1^+^), MP (Lin^–^cKit^+^Sca1^–^), common myeloid progenitors (Lin^–^cKit^+^Sca1^–^CD34^+^Fc^lo^), granulocyte-monocyte progenitors (Lin^–^cKit^+^Sca1^–^CD34^+^Fc^hi^), and megakaryocyte-erythroid progenitors (Lin^–^cKit^+^Sca1^–^CD34^–^Fc^lo^). (**H**) Colony formation assays from total FL cells. (**I**) Representative gating strategies for the Ki67/DAPI proliferation assay. E14.5 HSC cells were gated for G0 (Ki67^–^DAPI^–^), G1 (Ki67^+^DAPI^–^), and S/G2/M (Ki67^+^DAPI^+^) phases. (**J**) Quantification of percentage of fetal HSC, MPP, LSK, and MP cells in G0, G1, and S/G2/M phases. (**K**) Representative flow gating and quantification of intracellular ROS level in fetal HSCs (Lin^–^CD41^–^CD48^–^Mac1^+^CD150^+^cKit^+^Sca1^+^). (**L**) Seahorse assay to detect OCR in *Yy1^+/+^* and *Yy1^–/–^* FL LK cells. OCR was measured at baseline and after sequential treatment with oligomycin, FCCP, and rotenone/antimycin A. Number of fetuses = *n*; graphs show mean ± SD, *P < 0.05, **P < 0.01, ***P < 0.001, ****P < 0.0001, by unpaired 2-tailed *t* test (**B**, **D**, **F**, **G**, **H**, **K**, and **L**) and 2-way ANOVA (**G** and **J**).

Given that *Yy1^fl/fl^ Vav-Cre* neonates had a reduced number of all lineage blood cells (Figure 1J), we next evaluated how loss of YY1 affected HSC and progenitor numbers during fetal development. Grossly, *Yy1^fl/fl^ Vav-Cre* E14.5 fetuses were notably paler than the WT *Vav-Cre* control (Figure 2A). There was a small but statistically significant reduction of total FL cell number in *Yy1^fl/fl^ Vav-Cre* fetuses compared with the WT control (Figure 2B). Consistently, *Yy1^fl/fl^ Vav-Cre* FLs had reduced R1 (CD71^med^Ter119^–^) and R4 (CD71^med^Ter119^+^) erythrocytes (Figure 2, C and D). In addition, compared with *Vav-Cre* control mice, *Yy1^fl/fl^ Vav-Cre* mice had a significant reduction in both the percentage and absolute numbers of fetal E14.5 HSCs (Lin^–^CD41^–^CD48^–^Mac1^+^CD150^+^c-Kit^+^Sca1^+^), multipotent progenitors (MPPs, Lin^–^CD41^–^CD48^–^Mac1^+^CD150^–^c-Kit^+^Sca1^+^), LSKs (Lin^–^c-Kit^+^Sca1^+^), myeloid progenitors (MPs, Lin^–^c-Kit^+^Sca1^–^), common myeloid progenitors (Lin^–^c-Kit^+^Sca1^–^CD34^+^Fc^lo^), granulocyte-monocyte progenitors (Lin^–^c-Kit^+^Sca1^–^CD34^+^Fc^hi^), and megakaryocyte-erythroid progenitors (Lin^–^c-Kit^+^Sca1^–^CD34^–^Fc^lo^) (Figure 2, E–G). YY1-KO (*Yy1^fl/fl^ Vav-Cre*) E14.5 FL cells failed to give rise to CFU-GEMM, CFU-GM, BFU-E, and CFU-E, with significantly decreased colony numbers after plating in MethoCult M03434 complete medium (Figure 2H). Our data support the notion that YY1 is required for maintaining the HSC and progenitor cell pool during fetal development.

We next investigated how YY1 deficiency affects HSC proliferation and survival. Cell cycle status was assessed using Ki-67/DAPI staining. Within fetal HSC, MPP, LSK, and MP populations, cells were gated into G1 (Ki-67^+^DAPI^–^), S/G2/M (Ki-67^+^DAPI^+^), or G0 (Ki-67^–^DAPI^–^) phase (Figure 2I). YY1-deficient HSCs, MPPs, and LSKs exhibited a significant reduction in the proportion of cells in the G0 phase, indicating a loss of quiescence and increased proliferative activity (Figure 2J). Quiescent HSCs are known to maintain low intracellular ROS levels to preserve their long-term regenerative capacity (36, 37). Given that redox homeostasis is a critical regulator of HSC quiescence, we measured both intracellular and mitochondrial ROS levels. Compared with WT controls, YY1-deficient HSPCs displayed significantly elevated levels of both intracellular (Figure 2K) and mitochondrial ROS (Supplemental Figure 1A; supplemental material available online with this article; https://doi.org/10.1172/JCI188140DS1), despite similar mitochondrial mass between genotypes (Supplemental Figure 1B). Since mitochondria are a primary source of intracellular ROS, particularly through oxidative phosphorylation, increased ROS levels may reflect underlying mitochondrial dysfunction (38, 39). To assess mitochondrial function, we performed Seahorse XF Mito stress tests on LK FL cells from *Yy1^+/+^* and *Yy1^–/–^* fetuses. YY1-deficient cells showed significantly reduced oxygen consumption rates (OCRs) under both basal and maximal respiration conditions, along with decreased ATP production and spare respiratory capacity (Figure 2L). These findings indicate compromised mitochondrial function and bioenergetic stress in the absence of YY1. To evaluate whether YY1 deficiency affects the DNA damage response in fetal HSPCs, we measured γH2AX protein expression using Western blot in Lin^–^ FL cells (Supplemental Figure 1C) and flow cytometry in FL HSCs (Supplemental Figure 1D). No significant difference in γH2AX expression levels was observed between *Yy1^fl/fl^ Vav-Cre* and *Yy1^fl/fl^* HSPCs. To further assess whether YY1 deficiency affected fetal HSC apoptosis, we performed annexin V/DAPI staining in HSC, MPP, LSK, and MP populations (Supplemental Figure 1, E and G). There was no significant difference in the percentage of early or late apoptotic cells in HSC, MPP, and LSK populations between *Yy1^fl/fl^ Vav-Cre* and *Vav-Cre* fetuses, both in vivo (Supplemental Figure 1F) and ex vivo (Supplemental Figure 1H). Taken together, these results indicate that YY1 deficiency leads to elevated ROS levels and increased proliferation in fetal HSPCs, potentially driven by underlying mitochondrial dysfunction, without inducing apoptosis.

### YY1-deficient HSCs fail to repopulate the adult BM niche upon transplantation.

**Figure 3:**
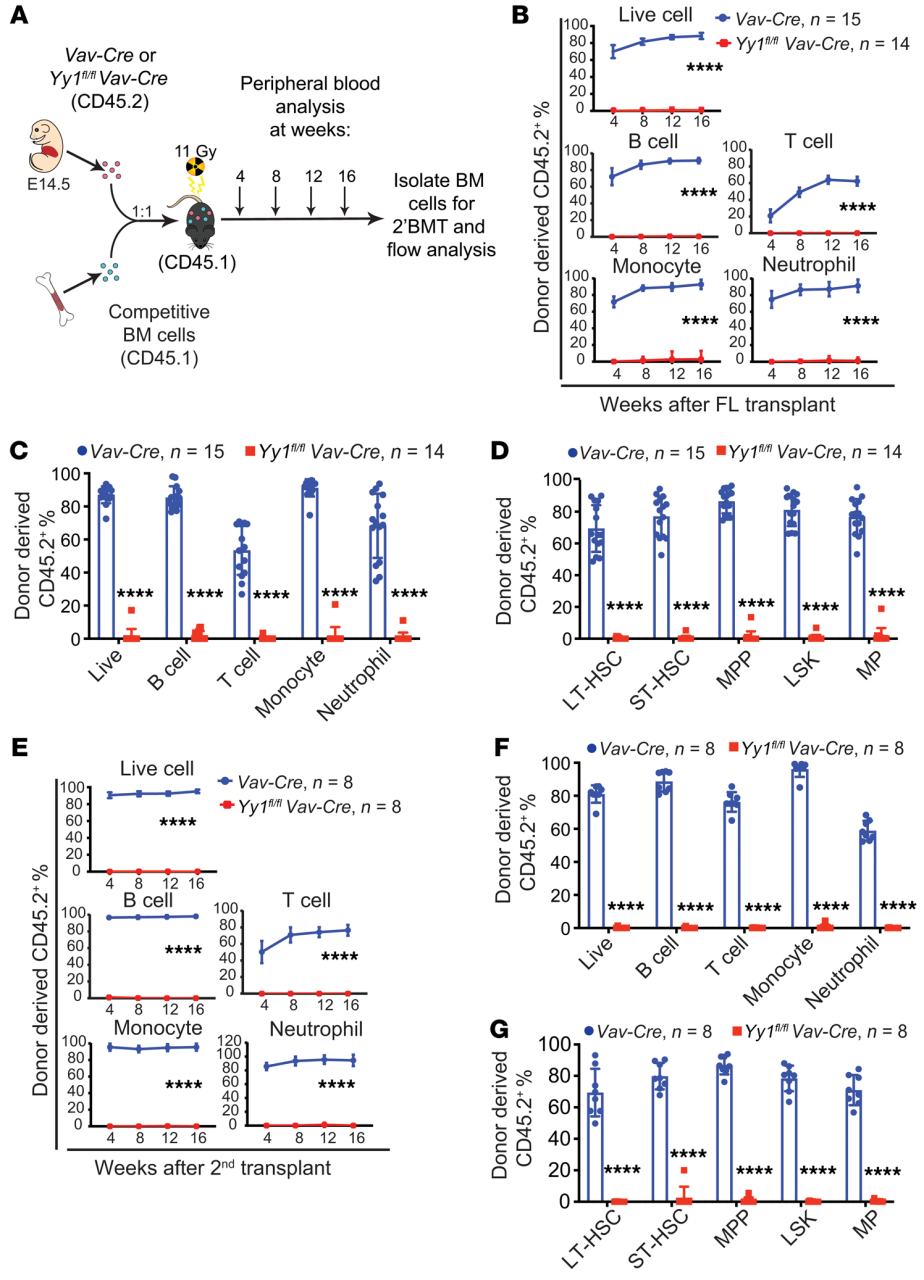
YY1-deficient HSCs fail to repopulate BM niche upon serial transplantation. (**A**) Experimental strategy: E14.5 *Vav-Cre* or *Yy1^fl/fl^ Vav-Cre* total FL cells were mixed with CD45.1^+^ BM competitor cells at a 1:1 ratio and transplanted into lethally irradiated CD45.1^+^ recipients. BM from reconstituted mice was harvested for analyses and for secondary BMTs at 16 weeks after transplantation. (**B**) Lineage evaluation of donor-derived contribution in peripheral blood live cells, B cells, T cells, monocytes, and neutrophils at 4, 8, 12, and 16 weeks after primary FL transplantation. (**C**) Donor-derived contribution of live cells, B cells, T cells, neutrophils, and monocytes in BM of recipient mice at 16 weeks after primary FL transplantation. (**D**) Donor-derived contribution in BM LT-HSC, ST-HSC, MPP, LSK, and MP after primary FL transplantation. (**E**) Lineage evaluation of donor-derived contribution in peripheral blood live cells, B cells, T cells, monocytes, and neutrophils at 4, 8, 12, and 16 weeks after second transplant. (**F**) BM donor-derived contribution at 16 weeks after second transplant. (**G**) Donor-derived contribution in BM LT-HSC, ST-HSC, MPP, LSK, and MP after second transplant. Number of mice = *n*; graphs show mean ± SD, ****P < 0.0001, by AUC measurement followed by Student’s *t* test (**B** and **E**) and 2-way ANOVA (**C**, **D**, **F**, and **G**).

Our previous study showed that YY1 is required for HSC long-term self-renewal in adult hematopoiesis (21). We therefore assessed the impact of YY1 deletion on fetal HSC self-renewal by competitive serial FL transplantation. Total FL cells from *Yy1^fl/fl^ Vav-Cre* and *Vav-Cre* fetuses were harvested at E14.5, mixed with freshly isolated WT CD45.1 BM cells, and then transplanted into lethally irradiated CD45.1 recipient mice. Lineage evaluations of peripheral blood were conducted every 4 weeks after transplantation (Figure 3A). Although donor-derived percentages, presented by the percentage of CD45.2^+^ cells (CD45.2%), in mice transplanted transplanted with *Vav-Cre* FL cells were in the range of 50%–80% of total live cells, B cells (Thy1.2^–^CD19^+^), T cells (Thy1.2^+^CD19^–^), neutrophils (Mac1^+^Gr1^hi^), and monocytes (Mac1^+^Gr1^–^) in peripheral blood, there were nearly no donor-derived cells detected in mice transplanted with *Yy1^fl/fl^ Vav-Cre* FL cells (Figure 3B). Similarly, at 16 weeks after transplantation, there were nearly no donor-derived B cells, T cells, neutrophils, monocytes (Figure 3C and Supplemental Figure 2A), LT-HSCs (Lin^–^Sca1^+^c-Kit^+^CD48^–^CD150^+^), ST-HSCs (Lin^–^Sca1^+^c-Kit^+^CD48^–^CD150^–^), MPPs (Lin^–^Sca1^+^c-Kit^+^ CD48^+^CD150^–^), or LSKs (Lin^–^Sca1^+^c-Kit^+^) (Figure 3D and Supplemental Figure 2B) detected in the BM of mice transplanted with *Yy1^fl/fl^ Vav-Cre* FL cells. On the contrary, mice transplanted with *Vav-Cre* FL cells showed approximately 50%–89% of donor-derived percentages (Figure 3D and Supplemental Figure 2B). Additionally, secondary transplantation of *Yy1^fl/fl^ Vav-Cre* cells was consistent with the primary transplantation results, revealing no donor-derived cells in the peripheral blood (Figure 3E), BM lineage cells (Figure 3F), and HSPCs (Figure 3G). Thus, our results showed that the YY1-deficient FL cells failed to reconstitute adult BM.

### YY1-deficient HSPCs fail to engraft the adult BM.

**Figure 4:**
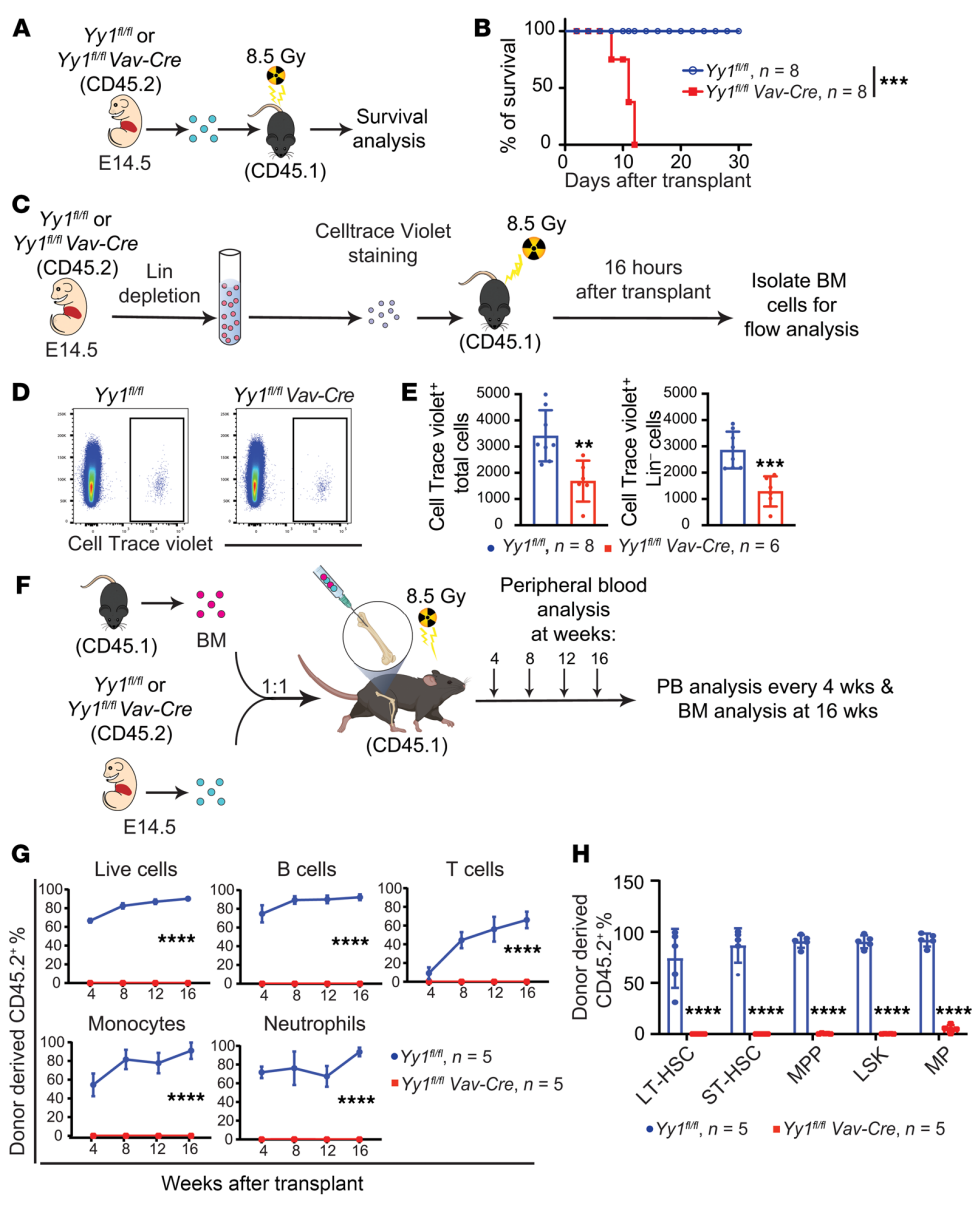
YY1 is essential for fetal HSC homing and engraftment at the BM niche. (**A**) Experimental strategy for noncompetitive FL cell transplantation: E14.5 *Yy1^fl/fl^* or *Yy1^fl/fl^ Vav-Cre* FL cells were transplanted into lethally irradiated CD45.1^+^ recipients without competitor. (**B**) Kaplan-Meier survival curve of mice transplanted with *Yy1^fl/fl^* versus *Yy1^fl/fl^ Vav-Cre* FL cells. (**C**) Experimental strategy for HSC homing experiment. E14.5 Lin^–^ FL cells were labeled with CellTrace violet and injected into the femurs of lethally irradiated recipient mice. BM cells from the recipient mice were harvested for evaluation within 16 hours after transplantation. (**D**) Gating strategy of CellTrace violet–positive cells. (**E**) Absolute cell numbers of CellTrace violet–positive total FL cells and Lin^–^ FL cells in the BM of recipient mice. (**F**) Experimental strategy for intraosseous FL cell transplant. (**G**) Donor-derived percentages of total FL cells, B cells, T cells, neutrophils, and macrophages in peripheral blood at 4, 8, 12, and 16 weeks after intraosseous transplant. (**H**) Donor-derived percentages of BM LT-HSC, ST-HSC, MPP, LSK, and MP at 16 weeks after intraosseous transplant. Number of mice = *n*; graphs show mean ± SD, **P < 0.01, ***P < 0.001, ****P < 0.0001, by simple survival curve analysis (Kaplan-Meier) (**B**), unpaired 2-tailed *t* test (**E**), AUC measurement followed by Student’s *t* test (**G**), and 2-way ANOVA (**H**).

Mature blood cell production is usually resumed at an interval of 2–3 weeks after transplantation (40). Because we found essentially no donor-derived cells in peripheral blood at 4 weeks after *Yy1^–/–^* FL transplantation (Figure 3B), we next assessed whether YY1-deficient FL stem progenitor cells are able to engraft the adult BM. We first conducted a noncompetitive transplant by transplanting either WT or YY1-deficient E14.5 FL cells into lethally irradiated recipient mice (CD45.1) without fresh BM competitors (Figure 4A). At 2 weeks after transplantation, all mice transplanted with E14.5 *Yy1^fl/fl^ Vav-Cre* FL cells died, whereas mice transplanted with FL cells from the WT littermates survived for at least 30 days after transplantation (Figure 4B). These data suggest that YY1-deficient FL cells fail to establish engraftment in the adult BM.

Homing of HSCs to the BM niche is a critical prerequisite for successful engraftment. To assess FL HSC homing, 1,000,000 E14.5 Lin^–^ FL cells from *Yy1^fl/fl^ Vav-Cre* and WT littermates (*Yy1^fl/fl^*) were labeled with CellTrace violet dye and transplanted into lethally irradiated recipient mice (Figure 4C). Over 99% of the transplanted cells were positive for CellTrace violet, confirming effective labeling (Supplemental Figure 3). Sixteen hours after transplantation, we quantified violet-positive total live cells and Lin^–^ cells in the BM of recipient mice (Figure 4D). Compared with controls, recipients of *Yy1^fl/fl^ Vav-Cre* cells showed a significant reduction in both total violet-positive live cells and violet-positive Lin^–^ cells, despite equivalent numbers of cells transplanted (Figure 4E). These result indicate that YY1-deficient Lin^–^ FL cells exhibit impaired migrating/homing to the adult BM. Given that approximately 40%–50% of YY1-deficient Lin^–^ FL cells were still able to reach the BM (Figure 4E), we next assessed whether YY1-deficient HSCs could engraft after direct injection into the BM cavity. Total WT or *Yy1^–/–^* FL cells were transplanted via intraosseous injection into the femurs of lethally irradiated CD45.1 recipient mice (Figure 4F). Mice receiving *Yy1^fl/fl^ Vav-Cre* cells showed no detectable donor-derived cells in peripheral blood or BM (Figure 4G and Supplemental Figure 4), whereas recipients of WT FL cells displayed more than 50% donor chimerism. Moreover, donor-derived LT-HSC, ST-HSC, LSK, MPP, and MP populations were nearly undetectable in mice transplanted with *Yy1^–/–^* cells, whereas these populations were readily detectable in WT recipients (Figure 4H). Together, these findings show that *Yy1^–/–^* FL stem progenitor cells exhibit impaired homing capacity and fail to establish engraftment in the adult BM niche.

### YY1 controls a genetic network governing fetal HSC migration, adhesion, and metabolism.

**Figure 5:**
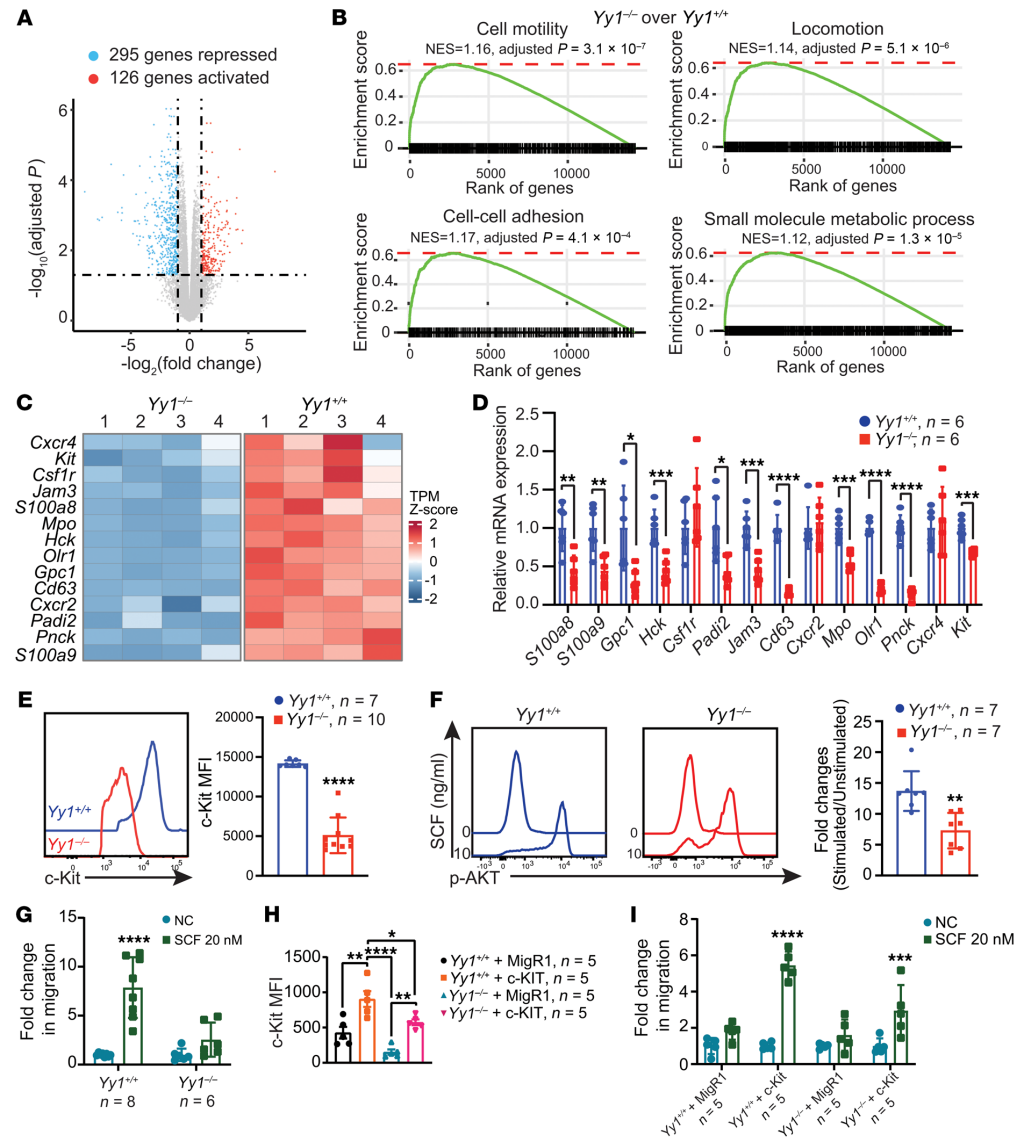
YY1 deficiency affects the genetic networks governing cell motility and metabolism. (**A**) Volcano plot showing the repressed and activated genes in *Yy1^–/–^* FL LK cells based on the fold change and adjusted *P* value. (**B**) GSEA: enriched biological processes shown with corresponding adjusted *P* values and normalized enrichment score (NES). (**C**) Heatmap depicting selected upregulated and downregulated genes involved in regulation of cell mobility, locomotion, and adhesion based on TPM. (**D**) qRT-PCR validation of marker gene expressions in FL LK cells. (**E**) Evaluation of c-Kit MFI in FL HSCs. (**F**) Phosphorylated flow analysis of AKT in FL LK cells and quantification of fold change in MFI of p-AKT. (**G**) Ex vivo migration assay of *Yy1^–/–^* and *Yy1^+/+^* Lin^–^ FL cells in response to SCF stimulation. (**H**) MFI of *c-Kit* in retrovirally infected FL Lin^–^ cells. (**I**) Ex vivo migration assay of *Yy1^–/–^* and *Yy1^+/+^* Lin^–^ FL cells infected with MigR1-c-Kit or MigR1 vector control. Number of fetuses = *n*; graphs show mean ± SD, *P < 0.5, **P < 0.01, ***P < 0.001, ****P < 0.0001, by unpaired 2-tailed *t* test (**D**–**F**), 2-way ANOVA (**G** and **I**), and 1-way ANOVA (**H**).

To gain mechanistic insights into YY1-mediated regulation of fetal HSC homing and engraftment, we performed RNA-Seq on Lin^–^c-Kit^+^ (LK) cells isolated by FACS from E14.5 *Yy1^fl/fl^* (*Yy1^+/+^*) and *Yy1^fl/fl^ Vav-Cre* (*Yy1^–/–^*) fetuses (Supplemental Figure 5A). Transcriptomic analysis identified 126 significantly upregulated and 295 downregulated genes in YY1-deficient cells (Figure 5A and Supplemental Figure 5B). Gene set enrichment analysis (GSEA) revealed that gene sets associated with cell motility, locomotion, adhesion, and metabolism were deregulated in *Yy1^–/–^* fetal LK cells (Figure 5B). Among the downregulated genes were several key regulators of cell migration and locomotion, including *S100a8*, *S100a9*, *Olr1*, *Gpc1*, *Mpo*, *Hck*, *Pnck*, *Jam3*, and *Padi2* (Figure 5C). These transcriptomic findings were further validated by quantitative real-time PCR (qRT-PCR) (Figure 5D). Notably, *Kit* transcript levels (Figure 5D) and c-Kit cell surface expression were both reduced in *Yy1^–/–^* fetal HSPCs compared with WT controls (Figure 5E). To determine whether YY1 deficiency impairs c-Kit signaling, we evaluated c-Kit–dependent phosphorylation of the downstream target AKT in response to SCF stimulation. Although SCF robustly induced AKT phosphorylation in WT fetal HSPCs, this response was significantly reduced in YY1-deficient cells (Figure 5F). Given the essential role of the SCF/c-Kit axis in HSC migration and adhesion (5, 41, 42) and observed homing defect in YY1-deficient HSPCs (Figure 4E), we next tested whether YY1 deficiency disrupts SCF-mediated fetal HSPC migration. In our ex vivo Transwell migration assay, WT FL HSPCs displayed enhanced migration in response to SCF stimulation, whereas YY1-deficient HSPCs failed to respond (Figure 5G). To test whether ectopic expression of c-Kit could rescue function defects caused by YY1 loss, including impaired SCF-mediated migration, hyperproliferation, and elevated mitochondrial ROS, we retrovirally expressed c-Kit in Lin^–^ FL cells from both WT and YY1-deficient fetuses. At 48 hours after infection, GFP+ HSPCs were analyzed for SCF-dependent migration capacity, cell cycle, and mitochondrial ROS levels. Ectopic c-Kit expression successfully restored c-Kit cell surface expression and rescued the SCF-mediated migratory response in YY1-deficient cells (Figure 5, H and I). However, c-Kit cell surface restoration did not rescue the hyperproliferative phenotype (Supplemental Figure 6, A and B) or elevated mitochondrial ROS levels (Supplemental Figure 6C) observed in YY1-deficient LK cells. Together, these results demonstrate that YY1 is essential for fetal HSPC function by regulating genes involved in migration, adhesion, and metabolism. Loss of YY1 impairs SCF/c-Kit signaling and abolishes SCF-mediated chemotactic responses, which can be rescued by restoring c-Kit expression.

### YY1 regulates chromatin accessibility at genes involved in cell adhesion and motility during fetal hematopoiesis.

**Figure 6:**
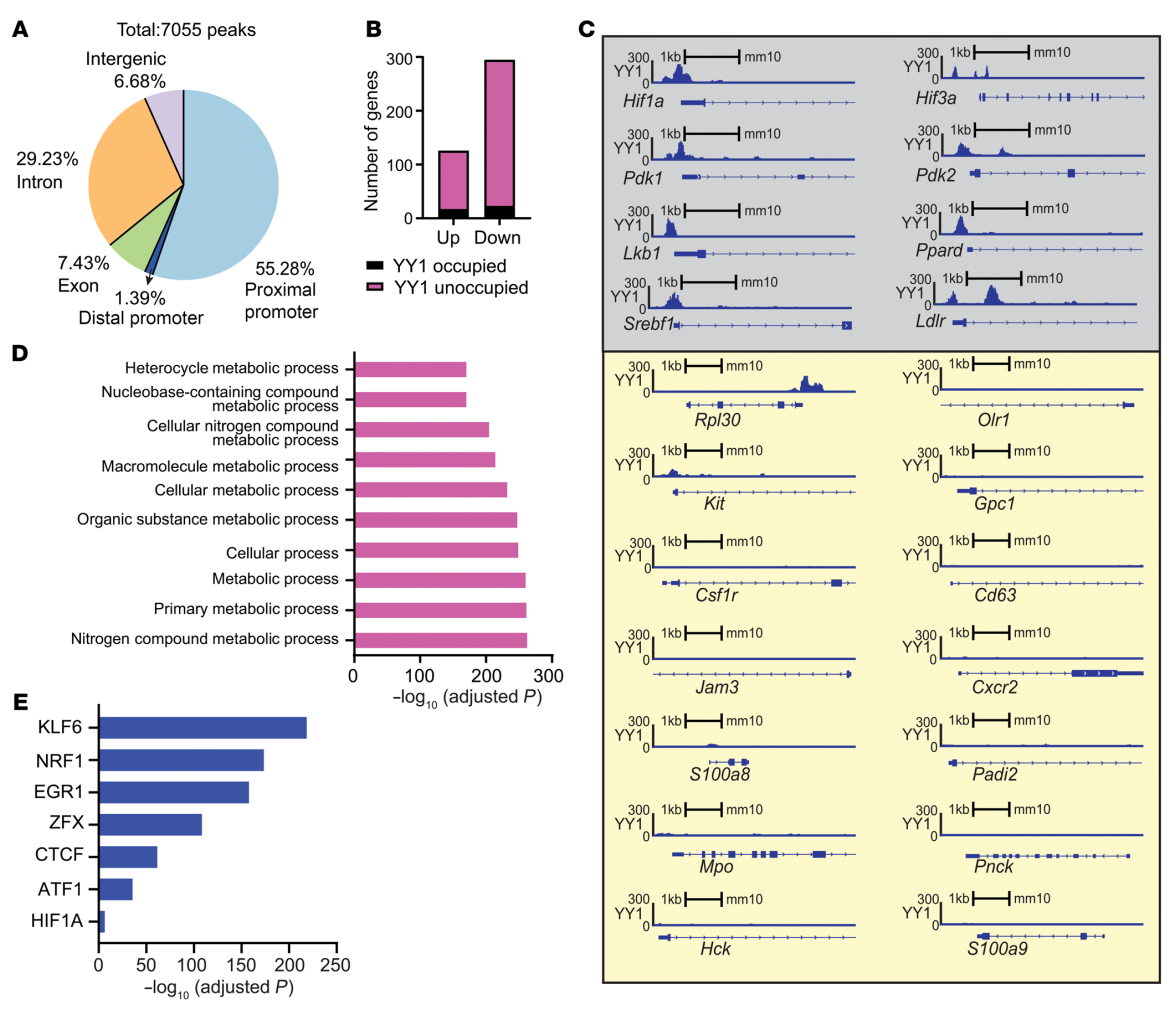
YY1-mediated direct and indirect regulations of fetal HSPCs. (**A**) Distribution of YY1 binding peaks at different genomic regions. (**B**) YY1 occupancies at upregulated and downregulated genes identified by RNA-Seq. (**C**) YY1 binds at the proximal promoters of genes involved in metabolism (gray, top) and no YY1 binding at the proximal promoters of genes involved in regulation of cell mobility, locomotion, and adhesion (yellow, bottom). *Rpl30* served as a positive control. (**D**) Top 10 overrepresented Gene Ontology terms on genes that have proximal promoters occupied by YY1. (**E**) YY1 occupies at the binding motifs of chromatin factors that are essential for chromatin reconstruction during HSC fetal-to-adult transition.

To determine whether YY1 controls fetal hematopoiesis through direct promoter binding and/or indirectly by regulating chromatin accessibility, we performed Cleavage Under Targets and Tagmentation (CUT&Tag) analysis for YY1 binding and ATAC-Seq analysis to evaluate chromatin accessibility in Lin^–^ FL cells. YY1 CUT&Tag analysis identified 7,055 YY1 binding peaks across various genomic regions, including proximal promoters, distal promoters, introns, exons, and intergenic areas (Supplemental Figure 5, C and D). Over half (55.28%) of YY1 binding peaks were located within proximal promoter regions compared with 29.23% in introns, 7.43% in exons, 6.68% in intergenic regions, and 1.39% in distal promoter regions (Figure 6A). Interestingly, YY1 binding was largely absent at the proximal promoters of most differentially expressed genes (DEGs) identified by RNA-Seq (Figure 5A). Specifically, YY1 did not occupy proximal promoters of 111 out of 126 upregulated genes and 274 out of 295 downregulated genes (Figure 6B and Figure 5A). Gene Ontology term analysis of genes with YY1 binding at proximal promoters highlighted an enrichment for pathways involved in metabolic process (Figure 6D). YY1 directly occupied the proximal promoters of key metabolic regulators, such as *Hif1a*, *Hif3a*, *Pdk1*, *Pdk2*, *Lkb1*, *Ppard*, *Srebf1*, and *Ldlr* (Figure 6C), indicating a direct role for YY1 in controlling metabolic gene expression during fetal hemopoiesis. In contrast, YY1 binding was not detected at the promoters of genes involved in cell adhesion, migration, or motility, such as *Olr1*, *Kit*, *Gpc1*, *Csf1r*, *Cd63*, *Jam3*, *Cxcr2*, *S100a8*, *Padi2*, *Mpo*, *Pnck*, *Hck*, and *S100a9* (Figure 6C), suggesting that YY1 regulates these processes largely through an indirect epigenetic mechanism. Motif enrichment analysis of YY1 binding regions revealed overlap with 94 transcription factor binding motifs, including those of master regulators of hematopoiesis, chromatin remodeling proteins, and factors that are essential for cell proliferation and stress response (Supplemental Figure 5E). Interestingly, YY1-bound regions were enriched for motifs bound by KLF6, NRF1, EGR1, CTCF, ZFX, ATF1, and HIF1A, factors known to orchestrate long-range chromatin interactions and regulatory loops during fetal-to-adult HSC transition (Figure 6E) (11, 12).

**Figure 7:**
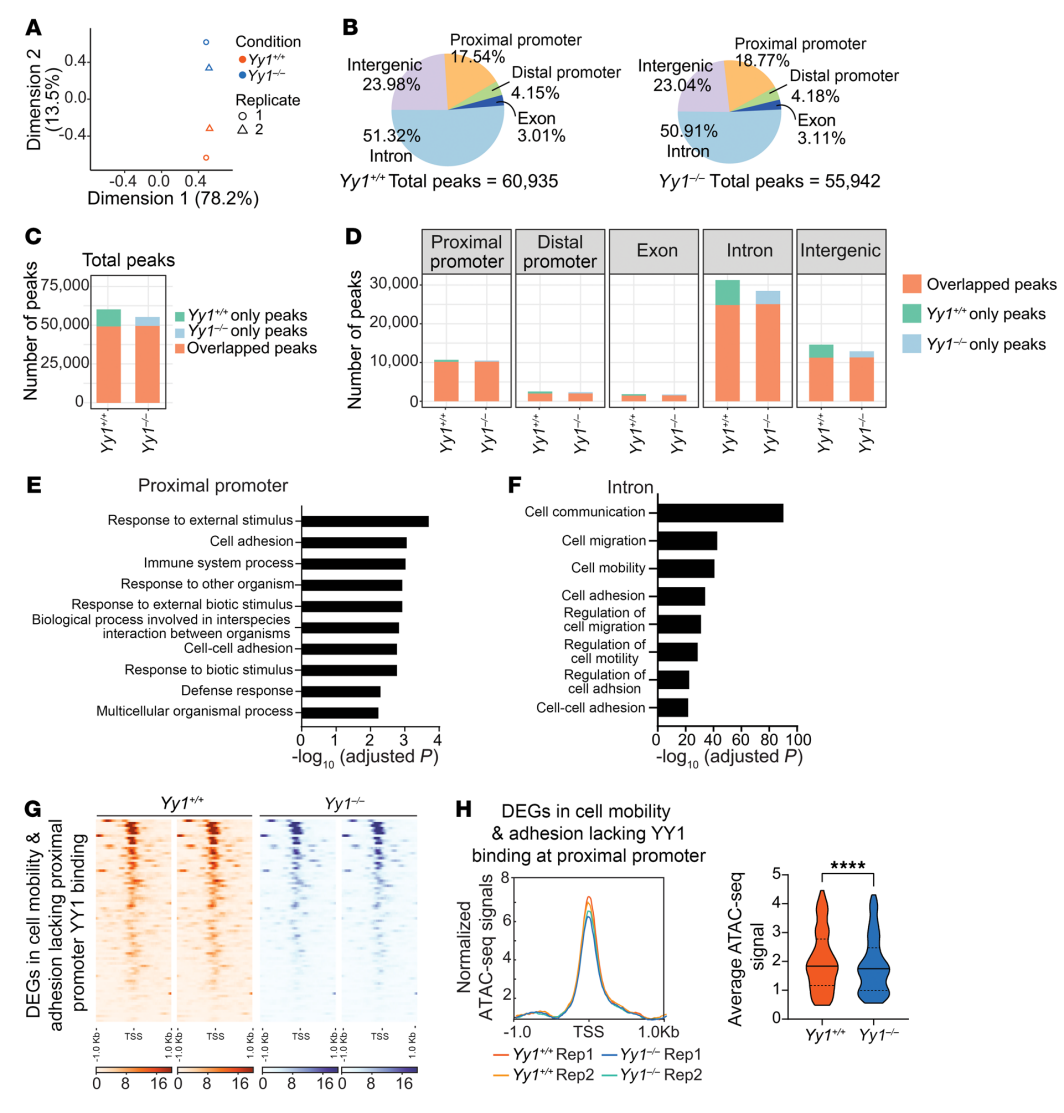
YY1 promotes chromatin accessibility at genes associated with cell mobility and adhesion pathways. (**A**) PCA of ATAC-Seq samples showing distinct clustering by genotype (*Yy1^+/+^* vs. *Yy1^–/–^*) in Lin^–^ FL cells. (**B**) Genomic distribution of accessible chromatin peaks in *Yy1^+/+^* and *Yy1^–/–^* Lin^–^ FL cells. (**C**) Total gain and loss of ATAC-Seq peaks in *Yy1^+/+^* and *Yy1^–/–^* conditions. (**D**) Genomic distribution of gain and loss of peaks. (**E** and **F**) Gene Ontology enrichment analysis of genes with loss of proximal promoter (**E**) and intron (**F**) ATAC-Seq peaks upon YY1 deletion. (**G** and **H**) ATAC-Seq signals at the proximal promoters of 81 DEGs involved in cell motility and adhesion pathways, which lack YY1 binding at these sites. (**G**) Heatmap showing reduced chromatin accessibility. (**H**) Average ATAC-Seq peak signals of each sample and quantification. ****P < 0.0001, by Wilcoxon matched-pairs signed rank test (**H**).

To investigate how YY1 regulates chromatin accessibility during fetal hematopoiesis, we performed ATAC-Seq analysis on E14.5 FL Lin^–^ cells from *Yy1^+/+^* and *Yy1^–/–^* fetuses. Principal component analysis (PCA) revealed distinct clustering between WT and YY1-KO samples, indicating substantial differences in chromatin accessibility (Figure 7A). A total of 60,935 ATAC-Seq peaks were identified in *Yy1^+/+^* cells, compared with 55,942 peaks in *Yy1^–/–^* cells. Both genotypes exhibited similar genomic distributions of chromatin accessible regions, with intronic regions being the most prevalent, followed by intergenic regions, proximal promoters, distal promoters, and exons (Figure 7B). YY1 deletion led to the loss of 11,146 peaks (~18% of the WT peak set), while 5,760 peaks (~10%) emerged in the KO samples, indicating an altered global chromatin accessibility upon YY1 loss (Figure 7C). Consistent with the global distribution, the majority of gained or lost peaks were located within intronic regions (Figure 7D). Promoter accessibility was relatively stable, with only approximately 4.6% (496/10,689) of lost peaks and approximately 2.6% (269/10,498) of gained peaks mapped to proximal promoters (Figure 7D). In YY1-KO cells, genes with reduced promoter and intron chromatin accessibility were significantly enriched for cell adhesion, motility, and migration pathways, consistent with our RNA-Seq findings (Figure 5B and Figure 7, E–H). Although YY1 did not bind the promoters of key adhesion and motility genes, the chromatin accessibility was altered upon YY1 deletion, suggesting that YY1 is required for a chromatin environment favorable for key transcription factor recruitment to these regions (Figure 6E). Altogether, these findings support a model in which YY1 regulates fetal hematopoiesis by directly binding to metabolic genes and modulating the chromatin landscape at other gene networks involved in cell mobility and adhesion.

### YY1 PcG function is necessary for fetal HSC long-term self-renewal.

**Figure 8:**
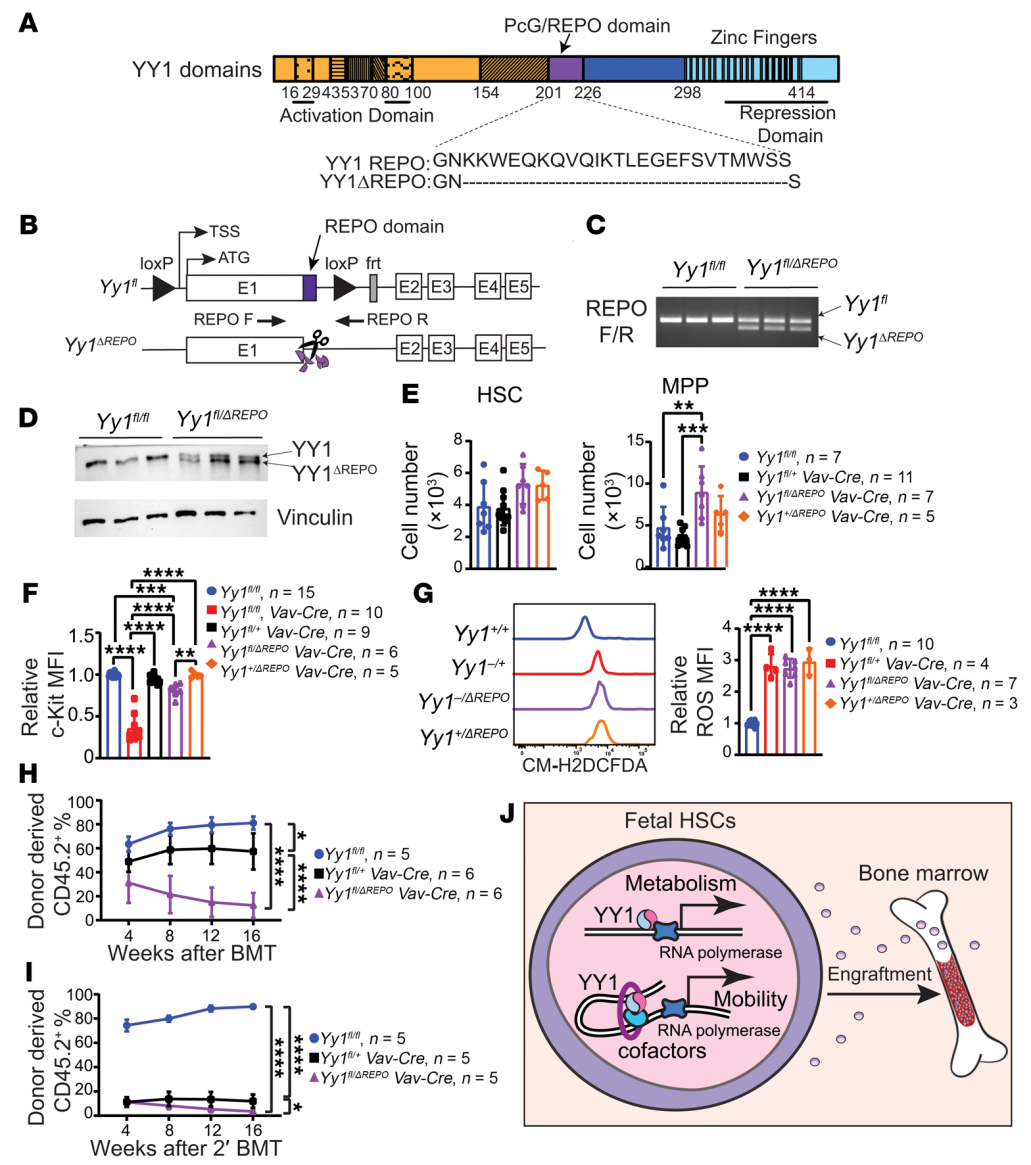
YY1 PcG function is essential for FL HSC reconstitution. (**A**) YY1 protein structural domains. (**B**) YY1Δ203-225 (YY1ΔREPO) generation by CRISPR/Cas9 method. (**C**) PCR genotyping confirms *Yy1^fl^* and *Yy1^ΔREPO^*. (**D**) Western blot of WT YY1 (upper band) and YY1ΔREPO (lower band). (**E**) Quantification of absolute cell numbers of HSC (Lin^–^CD41^–^CD48^–^Mac1^+^CD150^+^cKit^+^Sca1^+^) and MPP (Lin^–^CD41^–^CD48^–^Mac1^+^CD150^–^cKit^+^Sca1^+^). (**F**) c-Kit MFIs in HSCs. (**G**) Quantification of intracellular ROS level in fetal HSCs. (**H**) Lineage evaluation of donor-derived contribution in peripheral blood live cells at 4, 8, 12, and 16 weeks after primary and (**I**) secondary transplants. (**J**) YY1 regulates FL HSC metabolism and mobility through a dual mechanism: directly, by binding to gene promoters and regulating transcription, and indirectly, by interacting with essential cofactors to modulate chromatin accessibility. This regulatory network is critical for fetal HSC engraftment. Number of mice = *n*; graphs show mean ± SD, *P < 0.05, **P < 0.01, ***P < 0.001, ****P < 0.0001, by 1-way ANOVA (**E**–**G**) and AUC measurement, followed by 1-way ANOVA (**H** and **I**).

YY1 can recruit PcG complexes to DNA via a defined domain spanning amino acids 201–226, known as the YY1 REPO domain (43–45) (Figure 8A). This domain is critical for several YY1-mediated functions, including Vκ gene rearrangement in pro-B cells (46) and the survival of double-negative T cells (47). In addition, the YY1 REPO domain facilitates long-range chromatin remodeling and histone modification (43). To specifically assess the role of YY1’s PcG activity in fetal hematopoiesis, we generated YY1ΔREPO (*Yy1^+/ΔREPO^*) mice using CRISPR/Cas9 genome editing (Figure 8, B and C). The ΔREPO allele produces a YY1 protein that lacks PcG recruitment capability and fails to mediate H3K27me3 histone modification, while retaining all other known YY1 functions. The mutant protein is expressed at levels comparable to WT YY1 (Figure 8D), making this a powerful genetic model for dissecting YY1’s PcG-dependent functions in vivo (43, 44). To investigate the effect of YY1 REPO deficiency specifically in the hematopoietic system, *Yy1^+/ΔREPO^* mice were crossed with *Yy1^fl/fl^* mice to generate *Yy1^fl/ΔREPO^* mice, which were then crossed with *Yy1^fl/+^Vav-Cre* mice to generate *Yy1^fl/ΔREPO^ Vav-Cre* (*Yy1^–/ΔREPO^*) mice. In these mice, Cre recombinase expression deletes the WT allele of YY1, leaving the second allele with the germline REPO deletion. *Yy1^+/–^* mice were included as controls to assess the effects of YY1 haploinsufficiency. Flow cytometric analysis of E14.5 FL showed that the absolute numbers of HSCs (Lin^–^CD41^–^CD48^–^Mac1^+^CD150^+^cKit^+^Sca1^+^) were comparable among WT, *Yy1^–/ΔREPO^*, *Yy1^+/ΔREPO^*, and *Yy1*^+/–^ fetuses (Figure 8E). However, c-Kit surface expression was significantly reduced in *Yy1^–/ΔREPO^* HSCs compared with WT and *Yy1^+/ΔREPO^* cells, although the expression remained significantly higher than in *Yy1^–/–^* HSCs (Figure 8F). Additionally, intracellular ROS levels were elevated in both *Yy1^–/ΔREPO^* and *Yy1^+/ΔREPO^* HSCs (Figure 8G), although cell proliferation and SCF-dependent migration were not significantly affected (Supplemental Figure 7, A and B). Importantly, *Yy1^–/ΔREPO^* HSCs exhibited a profound defect in long-term self-renewal. In an FL transplantation assay, *Yy1^–/ΔREPO^* HSCs failed to maintain hematopoiesis beyond 8 weeks after transplantation (Figure 8H). In contrast, *Yy1^+/–^* HSCs supported initial engraftment but failed upon secondary transplantation (Figure 8I). Together, these findings demonstrate that the YY1 REPO domain is essential for the long-term self-renewal capacity of fetal HSCs. Thus, YY1 regulates fetal hematopoiesis through both its direct transcriptional activity and epigenetic functions, such as PcG-mediated regulation.

## Discussion

Our study underscores the essential role of YY1 in regulating fetal hematopoiesis, particularly in HSC migration and engraftment. We demonstrate that YY1-deficient FL HSCs fail to engraft in the adult BM niche after transplantation, highlighting a critical requirement for YY1 in this process. Given that FL HSC migration is fundamental for the sequential colonization of hematopoietic organs during development (29, 48), our findings position YY1 among the few transcription factors indispensable for this function. Furthermore, transcriptomic analysis reveals that gene networks governing cell locomotion and motility are selectively disrupted in YY1-deficient fetal HSPCs (Figure 5, A and B). As a multifunctional transcription factor and a core component of the PcG protein complex (43, 44, 49, 50), our findings suggest YY1 regulates fetal hematopoiesis through both direct transcriptional control and indirect epigenetic mechanisms. While YY1 directly regulates metabolic gene expression in fetal HSPCs, its impact on HSC migration and motility appears largely indirect, likely mediated through modulation of chromatin accessibility and facilitation of binding by other transcription factors (Figure 6C, Figure 7, and Figure 8J).

SCF and its tyrosine kinase receptor c-Kit are critical regulators of fetal hematopoiesis. Fetal HSCs express high levels of c-Kit throughout fetal development (51), and SCF/c-Kit signaling promotes both chemoattraction and retention of HSCs within the FL niche (5). Previous work from our group demonstrated that YY1 regulates SCF/c-Kit signaling in a context-dependent manner, with effects varying by cell type. Specifically, YY1 promotes c-Kit expression in adult HSCs (21) but does not affect c-Kit levels in early T cell progenitors (47). In line with its role in adult HSCs, our current study shows that YY1 deletion in fetal HSPCs results in reduced c-Kit mRNA levels (Figure 5D) and diminished c-Kit surface expression (Figure 5E). Although decreased c-Kit expression may contribute to an underestimation of HSPC frequencies in the YY1-KO group, functional analyses revealed that YY1-deficient HSPCs displayed impaired SCF/c-Kit signaling (Figure 5F) and a striking loss of responsiveness to SCF-mediated migration (Figure 5G). To further evaluate whether c-Kit downregulation is a causal factor underlying the observed functional defects in YY1-deficient HSPCs, we performed a c-Kit rescue experiment. Ectopic expression of c-Kit via retroviral transduction restored both c-Kit surface levels Figure 5H) and SCF-mediated migration in YY1-KO Lin^–^ FL cells (Figure 5I). Interestingly, despite rescuing the migration defect, c-Kit reexpression did not reverse the hyperproliferative phenotype or the elevated mitochondrial ROS levels associated with YY1 deletion (Supplemental Figure 7). These results suggest that while reduced c-Kit expression contributes to impaired migration, it is not the unifying cause of all phenotypic changes in YY1-deficient fetal HSPCs. In particular, YY1’s regulation of FL HSPC proliferation and mitochondrial homeostasis appears to involve c-Kit–independent mechanisms.

Although YY1 deletion during fetal development significantly reduced HSC numbers (Figure 2F), approximately 37% of YY1-deficient embryos survived until birth (Figure 1, E and F), indicating that residual HSC function can support partial fetal hematopoiesis. This contrasts sharply with YY1 deficiency in adult HSCs, which results in 100% lethality within 5–10 days after YY1 deletion (21). Importantly, *Yy1^–/–^* FL HSCs failed to engraft the adult BM niche upon transplantation (Figure 4, F–H), highlighting a critical YY1-dependent transition from fetal to adult hematopoiesis. To determine whether YY1 regulates distinct molecular programs at different developmental stages, we compared transcriptomic profiles from YY1-deficient fetal versus adult HSPCs. In FL LK cells, YY1 deletion resulted in 421 DEGs, whereas our prior RNA-Seq analysis of adult LSK cells showed 2,338 DEGs upon YY1 loss (NCBI’s Gene Expression Omnibus [GEO] GSE239743) (27). Strikingly, only 132 DEGs (5%) overlapped between fetal and adult datasets — 28 (3%) upregulated and 104 (6.1%) downregulated (Supplemental Figure 8A). GSEA revealed distinct pathway alterations: while both datasets showed deregulation in pathways related to secretion and myeloid activation (Supplemental Figure 8, B and C), cell motility pathways were uniquely affected in fetal HSPCs, whereas defense response and exocytosis pathways were specifically altered in adults. Despite differences in cell populations (fetal LK versus adult LSK cells) and experimental batches, these findings may indicate that YY1 regulates hematopoiesis in a developmental stage–specific manner and provide a framework for future studies aimed at dissecting YY1-dependent transcriptional programs and chromatin landscapes in phenotypically matched HSC populations across fetal and adult stages.

YY1’s role in long-range chromatin interactions also appears to be lineage specific, despite its ubiquitous expression (46, 52–56). Although chromosomal compartments and topologically associated domains are largely conserved between fetal and adult HSCs, intra–topologically associated domain interactions — especially enhancer-promoter contacts — are more dynamic during development. These interactions are mediated by developmental stage–specific transcription factors (11). We conducted transcription factor motif analysis of YY1 binding peaks in fetal HSPCs and identified a list of transcription factors that have been reported to be essential for enhancer-promoter interactions during fetal-to-adult HSC transition (Figure 6E). YY1 deletion in fetal Lin^–^ cells resulted in global changes in chromatin accessibility, including at promoters not directly bound by YY1 (Figure 7), suggesting that YY1 functions indirectly by organizing chromatin landscape and facilitating access of other key transcription factors.

To dissect the epigenetic role of YY1 in fetal hematopoiesis, we utilized a mutant mouse model lacking the YY1 REPO domain (YY1ΔREPO), which is required for PcG protein recruitment. YY1ΔREPO FL HSCs failed to sustain long-term self-renewal (Figure 8, H and I), demonstrating that YY1’s PcG-dependent function is essential for fetal hematopoiesis. Notably, YY1ΔREPO fetuses had normal HSC numbers (Figure 8E), suggesting that deletion of the REPO domain did not disrupt the overall folding and structural integrity of the YY1 protein. Interestingly, while YY1-KO FL HSCs failed to engraft entirely (Figure 3B and Figure 4, F–H), *Yy1^–/ΔREPO^* HSCs retained partial function and achieved approximately 30% donor chimerism after transplant (Figure 8H). Although *Yy1^–/ΔREPO^* HSCs expressed significantly higher c-Kit MFIs compared with YY1-KO cells (Figure 8F), these cells also displayed normal proliferation and SCF-mediated migration (Supplemental Figure 7), indicating that YY1 regulates fetal hematopoiesis through both REPO domain–dependent and REPO domain–independent mechanisms. Together, our findings support a model in which YY1 regulates fetal HSC function through a multifaceted mechanism involving direct transcriptional regulation at gene promoters for cell metabolism and through regulating chromatin accessibility at gene loci that are essential for cell migration and adhesion (Figure 8J). Loss of the REPO domain may disrupt higher-order chromatin organization and gene expression, impairing HSC long-term self-renewal function. Future studies leveraging the YY1ΔREPO model will be instrumental in delineating PcG-dependent versus PcG-independent roles of YY1 in developmental hematopoiesis.

## Methods

### Sex as a biological variable.

Both male and female fetuses were included in the study, and sex was not specifically identified as a biological variable during the experiments.

### Mice.

*Yy1^fl/fl^* mice (17, 57) in which the *Yy1* promoter region and exon 1 are flanked by loxP sites, were crossed with *Vav-Cre* mice to generate heterozygous *Yy1^fl/+^ Vav-Cre* mice. *Yy1^fl/+^ Vav-Cre* mice were then subsequently bred with *Yy1^fl/fl^* mice to generate homozygous *Yy1^fl/fl^ Vav-Cre* fetuses. *Vav-Cre* mice were bred with C57BL/6 mice to generate *Vav-Cre* fetuses. *Yy1^fl/fl^* mice were provided in-house (46). *Vav-Cre* mice were provided in-house (34). C57BL/6 mice were purchased from The Jackson Laboratory.

To generate *Yy1^+/∆REPO^* mice, 2 sgRNAs (5′-CACCGGACCCTGGAGGGCGAGTTCT-3′ and 3′-CCTGGGACCTCCCGCTCAAGACAAA-5′) flanking the REPO region were designed using CRISPR tools. In vitro–transcribed Cas9 mRNA and sgRNAs were microinjected into fertilized C57BL/6J zygotes, which were cultured to the 2-cell stage and implanted into pseudo-pregnant females at the Transgenic and Chimeric Mouse Facility at the University of Pennsylvania. Founder (F0) mice were genotyped by PCR and confirmed by Sanger sequencing. Germline transmission was established by breeding F0 mice with WT C57BL/6 mice, and F1 progeny were genotyped. Mice were backcrossed with C57BL/6 for 9 generations. To generate conditional deletion of the YY1 REPO domain, *Yy1^+/∆REPO^* mice were crossed with *Yy1^fl/fl^* mice to generate *Yy1^fl/∆REPO^* mice and then crossed with *Yy1^fl/+^ Vav-Cre* mice to generate *Yy1^fl/∆REPO^* *Vav-Cre* fetuses. *Yy1^+/∆REPO^* *Vav-Cre* mice were generated by crossing *Yy1^fl/+^ Vav-Cre* mice with *Yy1^+/∆REPO^* mice.

### Antibodies.

Fluorophore or biotin-conjugated antibodies were purchased from Thermo Fisher Scientific (eBioscience): CD3e (145-2C11), CD4 (RM4-5), CD5 (53-7.3) CD8 (53-6.7), CD41 (eBioMWReg30), B220 (RA3-6B2), Ter119 (TER-119), Gr1 (RB6-8C5), Mac1 (M1/70), IgM (eB121-15F9), CD19 (eBio1D3), CD93 (AA4.1), CD16/32 (mAb 93), CD127 (A7R34), CD45.2 (mAb104), CD45.1 (A20), Sca1 (D7), c-Kit (2B8), CD71(R17217), and Thy1.2 (53-2.1). CD48 (catalog 103427) and CD150 (TC15-12F12.2) were purchased from BioLegend. CD34 (RAM34) was purchased from BD Biosciences.

### Flow analysis.

FL cells from E14.5 embryos were harvested in IMDM with 2% FBS. The cells were spun down and resuspended in phosphate-buffered saline with 2% FBS before antibody staining (58). Cells used for HSC evaluation were subjected to Fc block for 15 minutes. Lineage markers for HS evaluation included a cocktail of biotin-conjugated antibodies against CD3e, CD4, CD5, CD8, CD41, Ter119, B220, CD19, and Gr1 with CD48. FL HSCs were defined as Lin^–^CD41^–^CD48^–^Mac1^+^CD150^+^c-Kit^+^Sca1^+^ cells (CD48 was included in biotin antibodies), and MPPs were defined as Lin^–^CD41^–^CD48^–^Mac1^+^CD150^–^c-Kit^+^Sca1^+^ cells. Lineage markers for progenitors included a cocktail of biotin-conjugated antibodies against CD3e, CD4, CD5, CD8, CD127, Ter119, B220, CD19, IgM, and AA4.1 (59). Cells were gated as LSK (Lin^–^c-Kit^+^Sca1^+^), MP (Lin^–^c-Kit^+^Sca1^–^), common myeloid progenitors (Lin^–^c-Kit^+^Sca1^–^CD34^+^CD16/32^lo^), granulocyte-monocyte progenitors (Lin^–^c-Kit^+^Sca1^–^CD34^+^CD16/32^hi^), and megakaryocyte-erythroid progenitors (Lin^–^c-Kit^+^Sca1^–^CD34^–^CD16/32^lo^). Erythrocytes were gated as R1 (CD71^med^Ter119^–^), R2 (CD71^hi^Ter119^–^), R3 (CD71^hi^Ter119^+^), R4 (CD71^med^Ter119^+^), and R5 (CD71^lo^Ter119^+^). The stained cells were acquired on BD LSR Fortessa or Cytek Aurora and data were analyzed by using FlowJo (version 10.5.3).

### Ki67 proliferation assay.

E14.5 FL cells were fixed with 4% PFA, permeabilized with 0.1% saponin in PBS, and stained with PE-conjugated Ki67 (BD Biosciences, 556027; 1:40 dilution) and DAPI solution (Thermo Fisher Scientific, 62248; 1:250 dilution) in addition to HSC, MPP, LSK, and MP markers as described above.

### Intracellular and mitochondrial ROS measurement.

Total FL cells from *Yy1^fl/fl^*, *Yy1^fl/fl^ Vav-Cre*, *Yy1^fl/ΔREPO^ Vav-Cre*, and *Yy1^+/ΔREPO^ Vav-Cre* fetuses at E14.5 were harvested and stained with HSC markers (Lin^–^CD41^–^CD48^–^Mac1^+^CD150^+^c-Kit^+^Sca1^+^). For the ROS measurement, cells were stained with either CellRox deep red dye (Invitrogen, C10422) at a final concentration of 500 nM in HBSS or cloromethyl-2′,7′-dichlorofluorescin diacetate (CM-H2DCFDA, Invitrogen, C6827) at a final concentration of 5 μM in PBS containing 2% FBS and incubated at 37°C for 30 or 15 minutes, respectively. The cells were washed once with HBSS and resuspended into the PBS containing 2% FBS and DAPI (1:500 or 1:50,000 dilution). The samples were acquired on the LSR Fortessa flow cytometer (BD Biosciences) or Cytek Aurora, and the ROS MFI was calculated by FlowJo (version 10.5.3). For mitochondrial ROS measurement, cells were stained with MitoSOX red (Invitrogen, M36008) at a final concentration of 5 μM in PBS containing 2% FBS and incubated at 37°C for 15 minutes. The samples were acquired on the Cytek Aurora, and the MitoSOX red MFI was calculated by FlowJo (version 10.5.3).

### Apoptosis assay.

Freshly isolated or serum-deprived overnight cultured FL cells from E14.5 fetuses were stained with surface markers as mentioned above to define HSC, MPP, LSK, and MP populations. Then, cells were resuspended in the binding buffer solution with FITC-conjugated anti–annexin V (BD Biosciences, 556419; 1:40 dilution). Next, cells were resuspended in DAPI solution (Thermo Fisher Scientific, 62248; 1:1,000 dilution). Samples were analyzed using the BD LSRII Fortessa, and results were analyzed by using FlowJo (version 10.0.7).

### Colony formation assay.

The colony formation assay was performed as described previously (21). The FL cells from E14.5 individual embryos were isolated in aseptic conditions and resuspended in IMDM with 2% FBS. The cells were plated in duplicate in MethoCult M3434 complete medium (StemCell Technologies) at a density of 2 × 10^4^ cells per 35 mm plate according to the manufacturer’s recommendation. After 10–12 days of culture at 37°C with 5% CO_2_, BFU-E, CFU-GM, and CFU-GEMM were identified and counted. CFU-E was counted after 2-day culture.

### Competitive transplantation of FL cells.

The total FL cells from *Vav-Cre*, *Yy1^fl/fl^*, *Yy1^fl/fl^ Vav-Cre*, *Yy1^fl/+^Vav-Cre*, and *Yy1^fl/ΔREPO^* *Vav-Cre* (CD45.2^+^) mice were freshly isolated at E14.5 and then mixed with freshly isolated BM cells from CD45.1^+^ mice at a 1:1 ratio. The C57BL/6 (CD45.1^+^) recipient mice were lethally irradiated with a Cesium source for a single dose of 11 Gy. Next, 10 × 10^5^ mixed cells were retro-orbitally transplanted to the recipient mice (59). The chimerism of peripheral blood was monitored every 4 weeks. The BM chimerism was detected at 16 weeks after transplantation. For secondary transplantation, 10 × 10^6^ BM cells from 2 donor mice were mixed and transplanted to lethally irradiated CD45.1^+^ congenic recipients at 16 weeks after primary transplantation as previously described (21).

### Noncompetitive transplantation of FL cells.

Total FL cells from *Yy1^fl/fl^* and *Yy1^fl/fl^ Vav-Cre* (CD45.2^+^) fetuses were isolated at E14.5. A total of 5 × 10^5^ cells were transplanted retro-orbitally into lethally irradiated (x-ray source, single dose of 8.5 Gy) C57BL/6 (CD45.1^+^) recipient mice.

### Intraosseous transplantation.

Total FL cells from *Yy1^fl/fl^* and *Yy1^fl/fl^ Vav-Cre* (CD45.2^+^) mice were isolated at E14.5 and then mixed with freshly isolated BM cells from CD45.1^+^ mice at a 1:1 ratio. The C57BL/6 (CD45.1^+^) recipient mice were lethally irradiated with an x-ray source, single dose of 8.5 Gy. Next, 5 × 10^5^ total cells were injected directly into the femur of recipient mice. The chimerism of peripheral blood was monitored every 4 weeks. BM chimerism was determined at 16 weeks after transplantation.

### Homing assay.

*Yy1^fl/fl^ Vav-Cre* and *Yy1^fl/fl^* FL cells were isolated from E14.5 fetuses. Lin^–^ cells were isolated by using the EasySep magnet system (StemCell Technologies) with antibodies against CD3, CD4, CD5, CD8, B220, CD19, Gr1, and Ter119 (eBioscience, antibody clone numbers listed above). One million Lin^–^ cells were stained with a final 5 μM concentration of CellTrace violet (Invitrogen, C34557) for 10 minutes at 37°C according to the manufacturer’s protocol. The stained cells were resuspended into PBS containing 2% mouse serum and transplanted retro-orbitally into lethally irradiated recipient (CD45.1) mice. BM cells from femurs, humerus, and tibia (6 total bones) were harvested within 16 hours after transplantation. CellTrace violet–positive cells were gated within live and Lin^–^ (CD3, CD4, CD5, CD8, B220, CD19, CD41, Gr1, and Ter119). The samples were acquired on an LSR Fortessa flow cytometer (BD Biosciences) and analyzed by FlowJo (version 10.5.3).

### qRT-PCR.

Lin^–^c-Kit^+^ cells were sorted from E14.5 FL and RNA was isolated by using QIAGEN RNeasy Mini Kit according to the manufacturer’s protocol. cDNA was synthesized with the SuperScript III First-Strand Synthesis System kit (Invitrogen) with oligo (dt) primers and was subjected to the qRT-PCR assay. Transcripts were detected by Roche Lightcycler96 with the cycle setting at 95°C (10 minutes), 95°C (10 seconds), 60°C (10 seconds), and 72°C (20 seconds) for a total of 40 cycles. *Gapdh* was used as an internal control for normalization, and quantification was determined by the ΔΔCt method. The primer sequences are included in Supplemental Table 1.

### Flow cytometry analysis of phosphorylated AKT.

E14.5 FL cells were deprived of serum and cytokines for 1 hour and then stimulated with murine SCF at 37°C for 10 minutes. The stimulated cells were fixed with paraformaldehyde at a final concentration of 2% (Electron Microscopy Sciences) at 37°C for 10 minutes. Surface proteins were detected with biotin-conjugated CD3e (145-2C11), CD4 (RM4-5), CD5 (53-7.3), CD8 (53-6.7), CDβ220 (RA3-6B2), Ter119 (TER-119), Gr1 (RB6-8C5), CD19 (eBio1D3), IgM (eB121-15F9), CD127 (A7R34), CD93 (AA4.1), and APC-eFluor780-conjugated c-Kit (2B8) antibodies from eBioscience. Phosphorylated AKT was detected by a primary antibody against p-AKT (Cell Signaling Technology, Ser473), followed by APC-conjugated donkey anti-rabbit F (ab′) 2 fragment (Jackson ImmunoResearch, 711-136-152). The samples were acquired on an LSR Fortessa flow cytometer (BD Biosciences) and analyzed by FlowJo (version 10.5.3).

### RNA-Seq and GSEA.

A total of 8 samples were chosen for RNA-Seq, 4 from *Yy1^+/+^* and 4 from *Yy1^–/–^* mice. Total RNA was purified from LK FL cells sorted from *Yy1^–/–^* versus *Yy1^+/+^* fetuses using RNeasy Micro kit (QIAGEN). Sequencing libraries were prepared by using Takara SMART-Seq V4 Ultra-Low Input according to the manufacturer’s specifications and were sequenced by an Illumina NovaSeq 6000 at the Gene Expression Center at University of Wisconsin Biotechnology Center. RNA-Seq reads were aligned by STAR (version 2.5.2b) to the mouse genome (version mm10) with GENCODE basic gene annotations (version M22). Gene expression levels were quantified by RSEM (version 1.3.0), and differential expression was analyzed by edgeR (version 3.36.0). A differentially expressed gene was required to have at least 2 fold-changes, an adjusted *P* value less than 0.05, and transcripts per million (TPM) of 1 or greater in all the replicates in at least 1 of the 2 conditions in comparison. GSEA was performed by fgsea (version 1.20.0) with gene sets from the Molecular Signatures Database (version 7.1 downloaded from https://bioinf.wehi.edu.au/MSigDB/). Adult RNA-Seq data (GEO GSE239743) (27) were analyzed by the same method as described above to identify differentially expressed genes and to perform GSEA.

### CUT&Tag analysis.

Lin^–^ E14.5 FL cells were isolated by using the EasySep magnet system (StemCell Technologies) with antibodies against CD3, CD4, CD5, CD8, CDβ220, CD19, IgM, CD127, AA4.1, and Ter119 (eBioscience, antibody clone numbers listed above). Around 300,000 Lin^–^ E14.5 FL cells were used for each CUT&Tag reaction with antibodies against IgG (Invitrogen, 0500C), H3K4me3 (Abcam, ab213224), and YY1 (Active Motif, 61780). The library was constructed following the published protocol (27, 60). To ensure reproducibility, 4 biological replicates were obtained to determine YY1 binding. CUT&Tag FASTQ files were first processed by removing the adapter CTGTCTCTTATACACATCT using the Cutadapt software (version 4.5) and then aligned to the mouse genome (version mm10) by Bowtie2 (version 2.5.1). Duplicated fragments were marked and removed by Picard (version 3.1.1). Peaks were called by MACS2 (version 2.2.9.1) for each replicate under a *q* value cutoff of 10^−6^. Peaks are defined as the genomic regions belonging to peaks called in all 4 replicates and with a minimum genomic span of 10 bp. Gene Ontology term overrepresentation was processed in the *goana* function.

### Western blot.

Whole cell lysates were prepared by lysing peripheral blood cells in 4× SDS sample buffer and were loaded onto 12% Mini-Protean TGX gels (Bio-Rad). Antibodies against YY1 (EPR4652, Abcam, ab109237) and Vinculin (E1E9V, Cell Signaling Technology, 13901), p-Histone H2A.X (Ser 139, Santa Cruz, sc-59803), and β-actin (AC-15, Sigma-Aldrich, A5441) were used according to the manufacturer’s recommendation.

### ATAC-Seq.

E14.5 Lin^–^ FL cells were isolated by negative selection using the EasySep magnet system (StemCell Technologies). ATAC-Seq libraries were prepared from 1 × 10^5^ cells using the ATAC-Seq kit (Active Motif, 53150), following the manufacturer’s instructions. Cells were incubated with Tagmentation Master Mix, and tagmented DNA was purified via DNA columns. Indexing was performed by 12-cycle PCR enrichment using Illumina UDP Indexes, followed by double-sided size selection (L = 1.1; R = 0.6) with Ampure beads (KAPA, Roche). Library size and quality were assessed on an Agilent 2100 Bioanalyzer with a high-sensitivity DNA kit and quantified by qPCR. Equimolar libraries were sequenced as 100 bp paired-end reads (PE100) on a NovaSeq X (Illumina), generating approximately 70 million fragments per sample. Library preparation and sequencing were performed at Institut de recherches cliniques de Montréal, Canada. Sequencing data were processed using the ENCODE ATAC-Seq pipeline (version 2.2.3) with alignment to the mm10 genome, peak calling, and signal calculation. Transcription factor motif enrichment was analyzed using CentriMo (MEME Suite version 5.3.3) with the HOCOMOCO v11 mouse motif database. Gene ontology enrichment was assessed with the *goana* function from *limma* (version 3.60.5).

### Seahorse assay.

Mitochondrial respiration was assessed using the Seahorse XF Mito stress test (Agilent, 103015-100) on an Agilent Seahorse XF Analyzer. LK cells from *Yy1^+/+^* and *Yy1^–/–^* E14.5 FLs were sorted using a BD Biosciences FACSAria and plated at 1.4 × 10^5^ cells/well on Cell-Tak–coated Seahorse XF microplates. Cells were incubated for 1 hour at 37°C in non-CO_2_ conditions in Seahorse XF Assay medium supplemented with 1 mM pyruvate, 2 mM glutamine, 3 mg/mL glucose, 1× penicillin-streptomycin, 100 ng/mL SCF, 10 ng/mL IL-6, and 5 ng/mL IL-3. OCR was measured at baseline and after sequential injections of oligomycin (2 μM), FCCP (2 μM), and rotenone/antimycin A (0.5 μM each). Mitochondrial parameters, including basal respiration, ATP production, maximal respiration, spare respiratory capacity, proton leak, and nonmitochondrial respiration, were calculated and normalized to cell number.

### In vitro migration assay.

E14.5 Lin^–^ FL cells were isolated via negative selection using the EasySep magnet system (StemCell Technologies) and incubated for 1 hour at 37°C with 5% CO_2_ in RPMI supplemented with 10% FBS. Migration assays were conducted using 24-well Transwell plates with 5 μm pore inserts (Costar/Corning). Lin^–^ cells (0.5 × 10^6^) were seeded in the upper chamber, and the lower chamber contained RPMI with 5% FBS and 20 μM SCF. After 5 hours of incubation, inserts were removed and Precision Count Beads (BioLegend) were added to the lower chamber to normalize migrated cells. Migrated cells were stained with lineage markers and quantified using a Cytek Aurora flow cytometer. Data were analyzed with FlowJo (version 10.5.3).

### Mitochondrial mass measurement.

Total E14.5 FL cells from *Yy1^fl/fl^* and *Yy1^fl/fl^ Vav-Cre* fetuses were harvested and stained with HSC markers (Lin^–^CD41^–^CD48^–^Mac1^+^CD150^+^c-Kit^+^Sca1^+^). For mitochondrial mass measurement, cells were stained with warm MitoTracker deep red FM dye (Invitrogen, M46753) at a final concentration of 10 nM in PBS containing 2% FBS and incubated at 37°C for 20 minutes. The cells were washed once with PBS containing 2% FBS and resuspended into the PBS containing 2% FBS and DAPI (1:50,000 dilution). The samples were acquired on the Cytek Aurora and the MitoTracker deep red MFI was calculated by FlowJo (version 10.5.3).

### Flow cytometry analysis of γH2AX.

Total FL cells from *Yy1^fl/fl^* and *Yy1^fl/fl^ Vav-Cre* fetuses at E14.5 were stained with HSC markers (Lin^–^CD41^–^CD48^–^Mac1^+^CD150^+^c-Kit^+^Sca1^+^). Cells were then fixed and permeabilized using eBioscience Foxp3/Transcription Factor Staining buffer set (Invitrogen, 00-5523-00) following the manufacturer’s recommendations. Fixed cells were incubated with PE anti-H2A.X Phospho (Ser139) antibody (2F3, BioLegend, 613411) for 30 minutes in permeabilization buffer (1:200 dilution). Cells were washed twice with permeabilization buffer and resuspended into the PBS containing 2% FBS. The samples were acquired on the Cytek Aurora, and the γH2AX MFI was calculated by FlowJo (version 10.5.3).

### Statistics.

All data are expressed as mean ± SD. All statistical analyses were conducted using GraphPad Prism unless otherwise specified. Differences between the groups were determined using a 2-tailed Student’s *t* test when comparing 2 groups or a 1-way ANOVA followed by Tukey’s post hoc test when comparing 3 or more groups. Two-way ANOVA followed by Tukey’s post hoc test was used to compare the changes in the proportion of cells undergoing apoptosis, as measured by annexin V and DAPI flow cytometric analysis, and to compare cells in different phases of the cell cycle, as measured by Ki67 and DAPI flow cytometry analysis. The AUC measurement was performed for line graphs and followed by 2-tailed Student’s *t* test (for 2 cohorts) or 1-way ANOVA (for 3 or more cohorts). Simple survival curve analysis (Kaplan-Meier) was performed to compare survival differences between cohorts. The Wilcoxon matched-pairs signed rank test was performed to compare the ATAC-Seq average signals at proximal promoter regions between cohorts. χ^2^ analysis was used to compare expected and observed offspring counts obtained from the breeding between *Yy1^fl/+^ Vav-Cre* and *Yy1^fl/fl^* mice at different stages using Microsoft Excel. *P* values of 0.05 or less were considered statistically significant.

### Study approval.

All experiments involving animals were approved by the IACUC of the University of Wisconsin-Madison and the University of Pennsylvania and conform to the appropriate regulatory standards.

### Data availability.

The data supporting the findings of this study can be found within the article and its supplemental materials. The RNA-Seq, CUT&Tag, and ATAC-Seq data generated in this study were deposited in the NCBI’s GEO under the accession numbers GSE279864, GSE279863, and GSE298778, respectively. Values for all data points in graphs are reported in the Supporting Data Values file. Additional information needed to reanalyze the data presented in this paper is available upon request.

## Author contributions

XP designed experiments. ZL, SS, YW, DKS, TRA, XY, JPL, CGP, and ALFVA performed experiments. ZL, SS, YW, DKS, PL, TRA, ALFVA, XY, MB, JZ, MLA, CGP, and XP analyzed and interpreted the data. ZL, SS, YW, PL, MLA, and XP wrote the manuscript. The order among co–first authors is based on the relative equal contribution made by each author to the manuscript.

## Supplementary Material

Supplemental data

Unedited blot and gel images

Supporting data values

## Figures and Tables

**Table 1:**
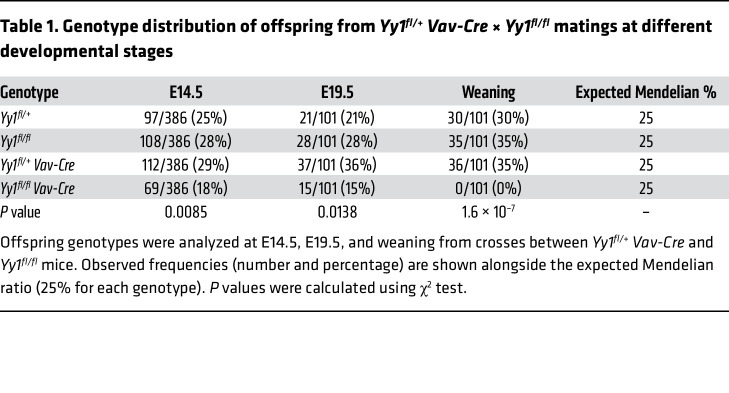
Genotype distribution of offspring from *Yy1^fl/+^ Vav-Cre* × *Yy1^fl/fl^* matings at different developmental stages

## Version 1 07/30/2025

In-Press Preview

## Version 2 10/01/2025

Electronic publication
