# Supplementary material for: Chromatin factor YY1 controls fetal hematopoietic stem cell migration and engraftment in mice
Source: J Clin Invest. 2025 Jul 29;135(19):e188140. doi: 10.1172/JCI188140 (PMC12483607; doi:10.1172/JCI188140)
Supplement: Supplemental data [file jci-135-188140-s193.pdf]

Supplementary data

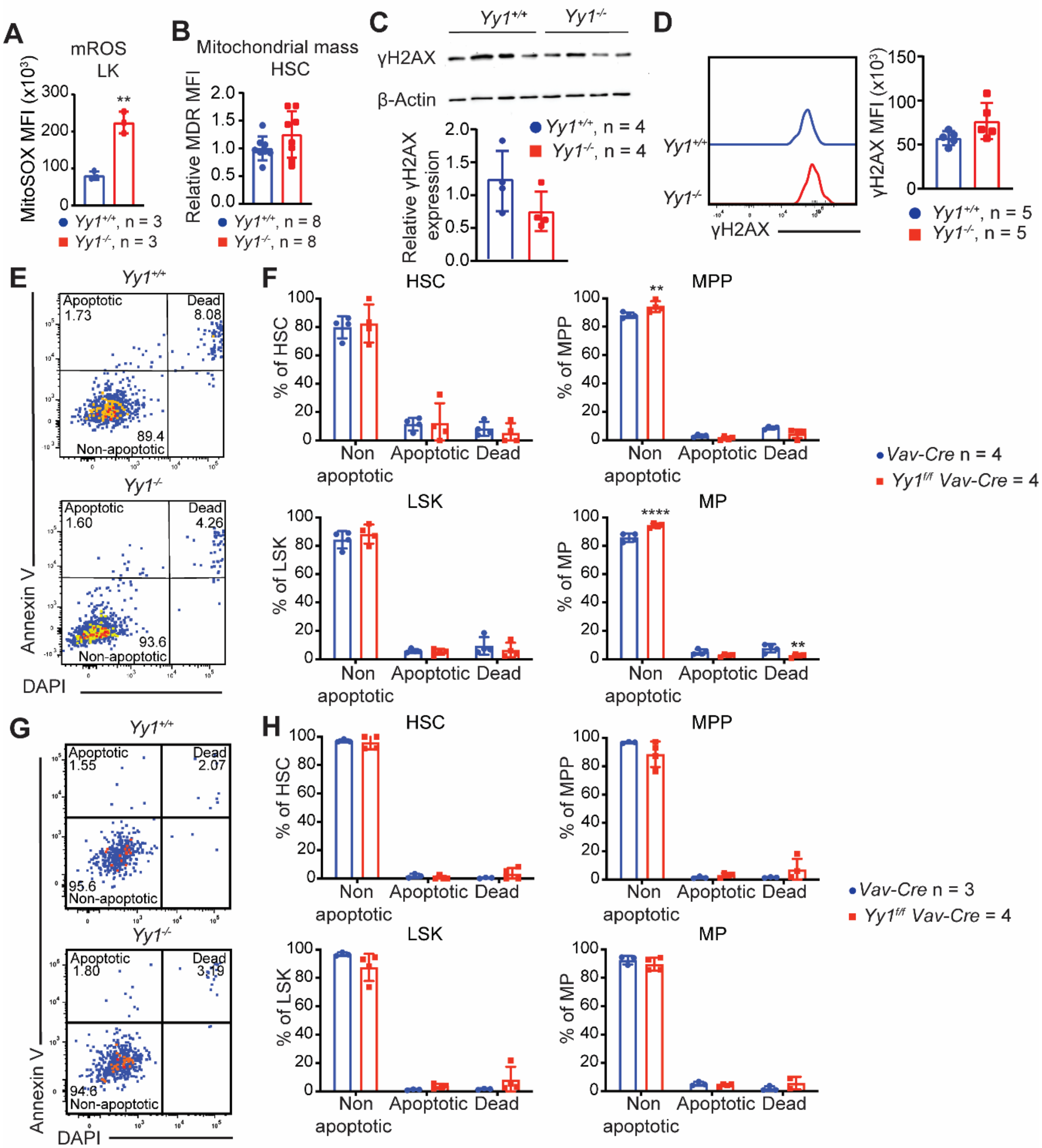

Supplemental Figure 1. Flow cytometry evaluation of (**A**) Mitochondrial ROS levels and (**B**) Mitochondrial mass in WT and YY1 KO HSCs. (**C**) WB and quantification of γH2AX in Lin⁻ FL cells. (**D**) Flowcytometry evaluation of γH2AX expression in WT and YY1 KO HSCs. **(E-H)** In vivo and ex vivo survival assay of fetal HSPCs**. (E)** Representative flow gating strategy for non-

apoptotic (Annexin V^-, $DAPI^-$), apoptotic (Annexin V^+, $DAPI^-$) and dead cells (Annexin V^+, $DAPI^+$) in the freshly isolated FL HSPC cells. **(F)** Quantification of percentage of non-apoptotic, apoptotic, and dead cells in FL HSC, MPP, LSK, and MP cells. **(G)** Representative flow gating strategy and (**H**) quantification of non-apoptotic, apoptotic, and dead FL HSPCs cultured with serum free media overnight. N represents the number of fetuses; graphs show means ± SD, **P <0.01, ****<0.0001 by unpaired t test (**A-D**), 2-way ANOVA (**F** and **H**).

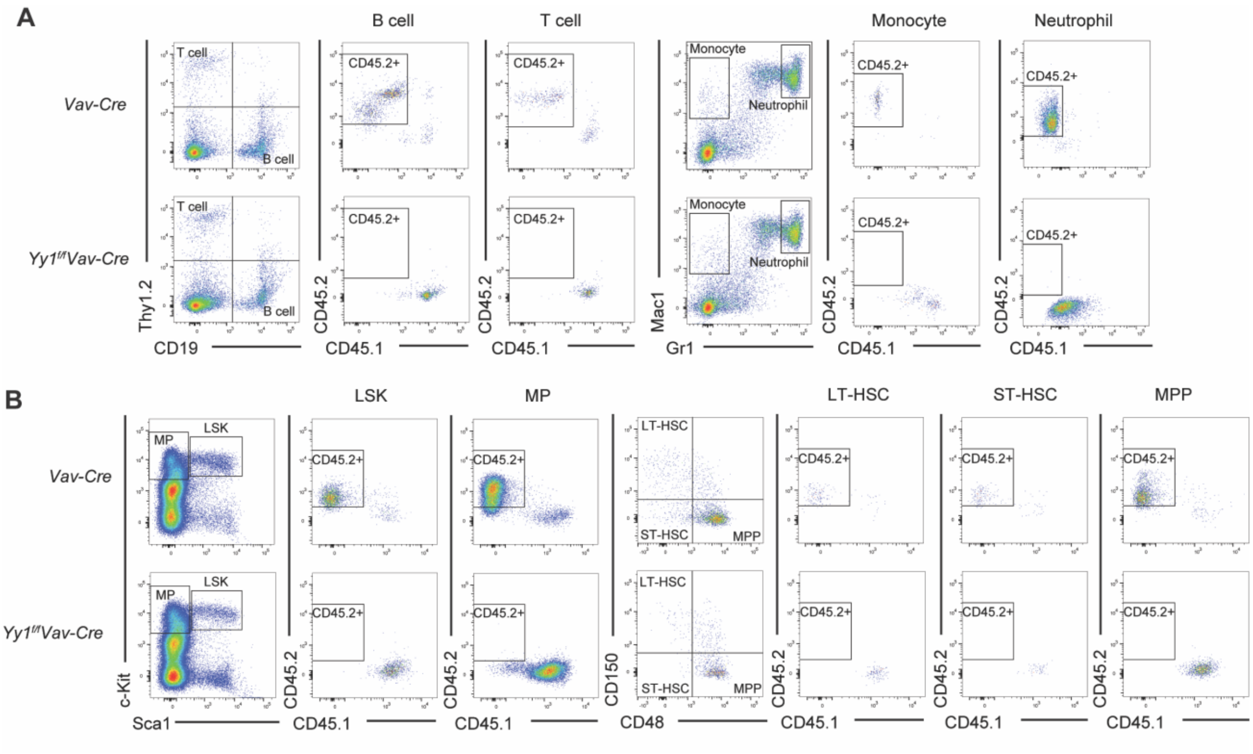

Supplemental Figure 2. (A) Representative flow gating strategy for donor derived BM B cells (CD45.2^{+}Thy1.2^{-}CD19^{+}), T cells (CD45.2^{+}Thy1.2^{+}CD19^{-}), neutrophils (CD45.2^{+} Mac1^{+}Gr1^{hi}) and monocytes (CD45.2^{+} Mac1^{+}Gr1^{-}) of FL transplant. (**B**) Representative flow gating strategy for donor derived BM LT-HSC (CD45.2^{+}Lin^{-}Sca1^{+}c-Kit^{+}CD48^{-}CD150^{+}), ST-HSC (CD45.2^{+}Lin^{-}Sca1^{+}c-Kit^{+}CD48^{-}CD150^{-}), MPP (CD45.2^{+}Lin^{-}Sca1^{+}c-Kit^{+}CD48^{+}CD150^{-}), LSK (CD45.2^{+}Lin^{-}Sca1^{+}c-Kit^{+}) and MP (CD45.2^{+}Lin^{-}Sca1^{-}c-Kit^{+}) populations.

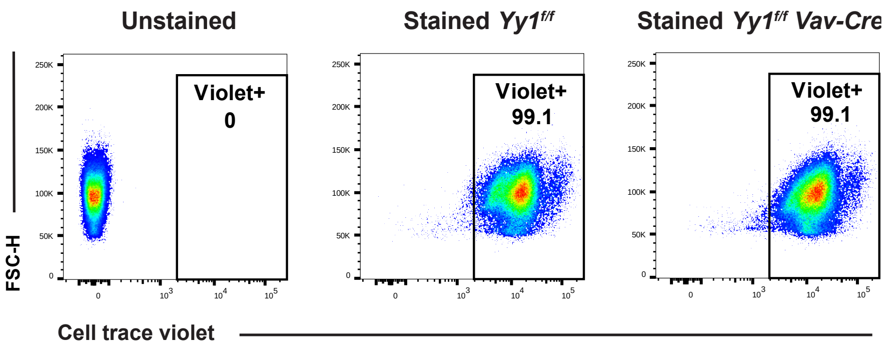

Supplemental Figure 3. CellTrace Violet dye labeled over 99% cells. Representative flow grating strategy for percentage of CellTrace Violet^{+} *Yy1$^{f/f}$* and *Yy1$^{f/f}$ Vav-Cre* Lin- fetal liver cells after staining.

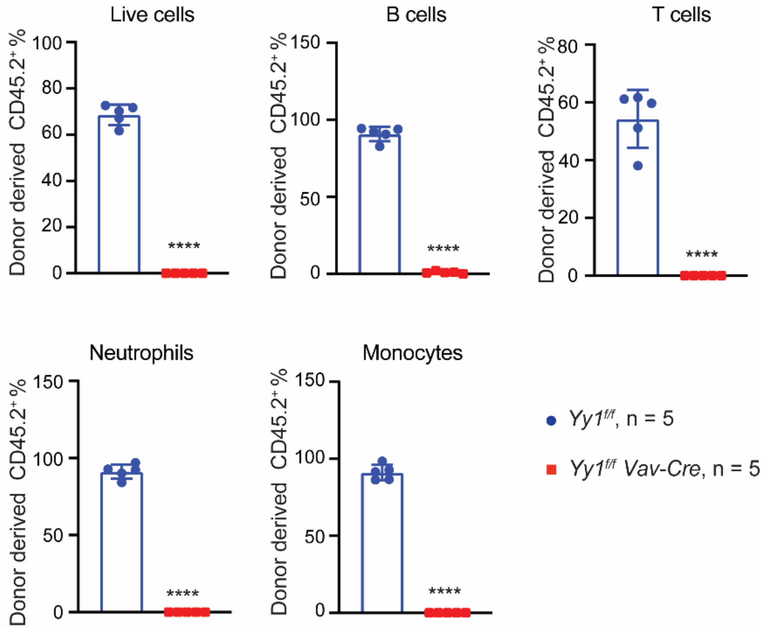

Supplemental Figure 4. Donor derived percentage of total live cells ($CD45.2^+$), B cells ($CD45.2^+Thy1.2^-CD19^+$), T cells ($CD45.2^+Thy1.2^+CD19^-$), neutrophils ($CD45.2^+Mac1^+Gr1^{hi}$) and monocytes ($CD45.2^+$ $Mac1^+Gr1^-$) in bone marrow of recipient mice that received intraosseous transplant of wild-type or YY1 deficient FL cells**.** N represents the number of mice; graphs show means ± SD, ****P<0.0001 by unpaired t test.

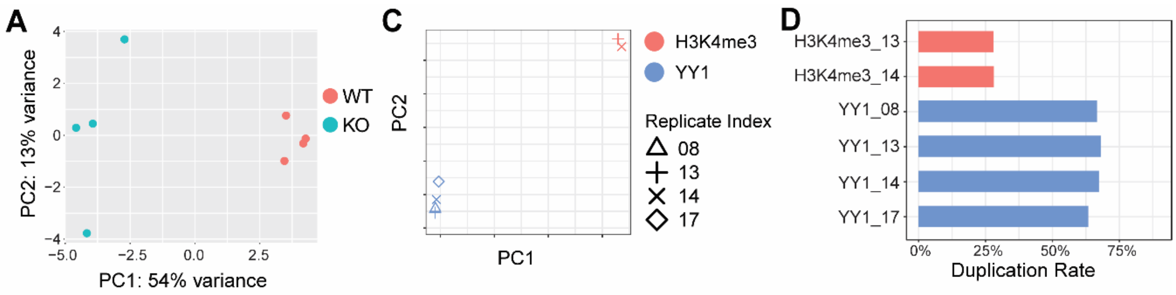

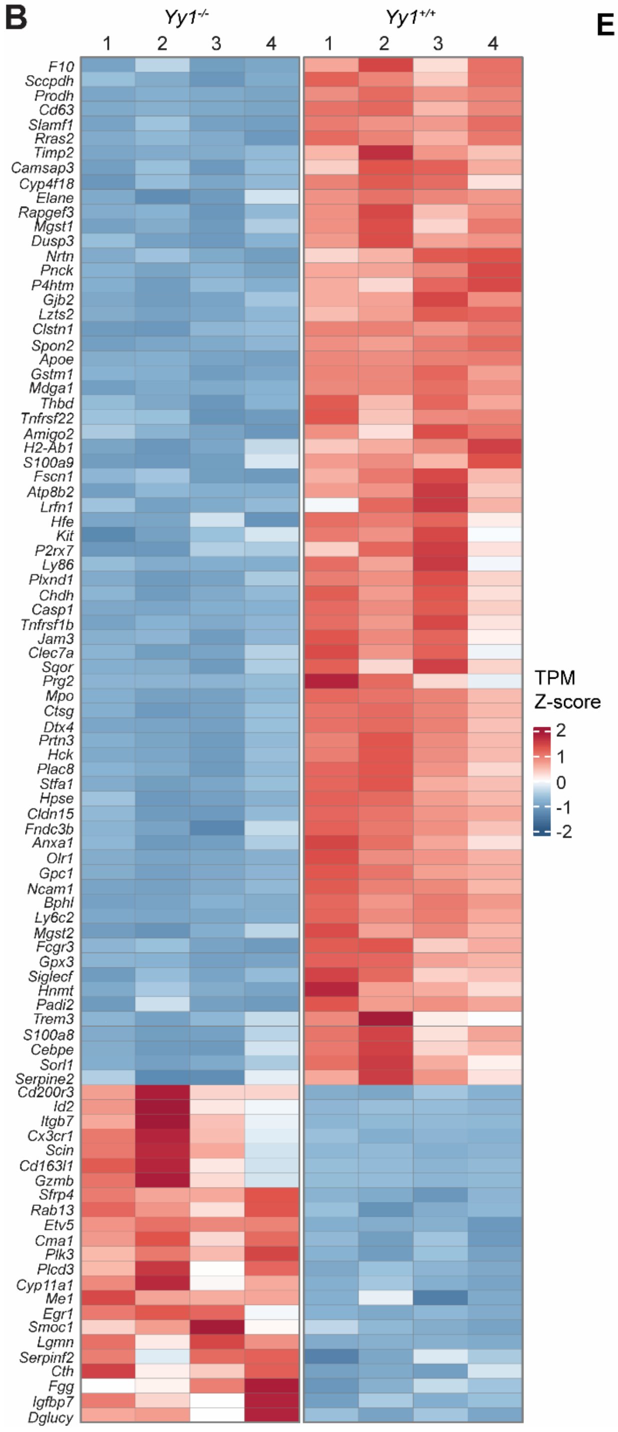

E

Rank	TF	Adjusted P	Motif Sequence
1	KLF3	0	GGRGVDGGGCGGGGCYGGG
2	E2F4	0	RGGGGGCGGGRMNN
3	SP3	0	SGVVGGGGGCGGGGCBRGSS
4	SP2	0	GGSSVGGGGGCGGGGCCDGSGS
5	KLF1	2.4E-303	DGGGYGKGGCYGGG
6	E2F1	3.8E-272	GRGGGCGGGRMV
7	KLF5	2.1E-269	RGGGSWGGGHGGGGC
8	SP4	1.5E-259	SRGVARGRGGCGGRGCHDRR
9	KLF4	3.7E-228	KRRRVWGGGTGKGGC
10	KLF6	2.9E-219	GSRRGGGHGGGGMHGGGRV
11	FOXI1	2.7E-188	YTBTGATTGGYY
12	SP1	1.1E-184	GGVGGGGGVGGGGGMGGGGCSGGG
13	NRF1	3.1E-174	CWSTGCGCATGCGCVRS
14	EGR1	1.4E-158	GKGCGKGGGCGGGG
15	NFYB	3.1E-154	YRRCCAATSAGMR
16	KLF15	9.4E-147	RGGGMGGRGVDGGGGGRGG
17	ZBT17	1.7E-140	GGSGGHGGGGGAGGGGMGG
18	NFYC	4.3E-140	BYARCCAATCAGAR
19	EGR2	6.8E-119	KGHGTGGGHGGGGG
20	E2F2	1.5E-109	GGCGCGAAAC
21	ZFX	3.5E-109	GSCBVGGCCTGSSSSSCBS
22	E2F3	1.3E-98	GGGCGGGARS
23	CTCFL	4.4E-97	GCCRSCAGGGGGCGCYVB
24	NFYA	1.4E-96	BYRRCCAATCAGAR
25	WT1	1.3E-94	RGGVGGGGGAGGRGGVGGRG
26	E2F7	4.4E-93	GDGGCGGGAARDR
27	SP5	1.6E-86	RRGGRGGRGGRGRRGGGGGAGGRG
28	MBD2	4.0E-77	SSGKCCGGMGR
29	FLI1	9.6E-74	GGVRCCGGAAGYGS
30	E2F6	3.6E-71	NKGGCGGGARRG
31	MECP2	6.0E-71	SCCGGRR
32	GABPA	1.2E-69	GGVRCCGGAAGTGV
33	ELF1	1.4E-69	SRACCCGGAAGTGS
34	CTCF	2.5E-62	YGGCCASCAGRGGGCGCYVB
35	SALL4	2.2E-60	GSDGGGWGGG
36	TYY1	2.1E-59	CAAVATGGCGGC
37	ZN281	1.9E-54	GGGGTGGGGGAGGGG
38	AP2B	1.3E-49	GCCYGVGGGS
39	PBX3	1.7E-48	YBYTGATTGRYTGR
40	ELK1	1.4E-47	RCCGGAAGTGV
41	ARNT	3.6E-46	BYRCGTGC
42	AHR	6.0E-46	DTYGCGTGM
43	ELK4	3.0E-43	SRRCCGGAAGYRG
44	FEV	2.6E-41	GCVGGAAGYG
45	TFEB	2.9E-38	RGTCACGTG
46	MAZ	5.0E-37	GGGGGAGGGGGDGRGRRRGRRG
47	ATF1	3.3E-36	VTGACGTCAS
48	ZFP57	2.2E-34	CTGCGGCARR
49	AP2C	3.0E-34	YRSCCYCAGGSYN
50	MYC	3.3E-34	MGCCACGTGSBC
51	ETS1	5.0E-34	VRRRCMGGAAGTGG
52	KAISO	4.5E-32	SARRYCTCGCGAGAV
53	TAF1	4.5E-31	VAARAWGGCGGMGG
54	MAX	2.4E-30	SVSCACGTGGC
55	AP2A	6.1E-30	VTRSCCTGRGGC
56	PKNX1	8.6E-30	TGABTGRCAGS
57	USF2	9.3E-27	GGGTCACGTGRBSVSSSSB
58	RFX1	5.2E-26	SSSSCBGTTGCCAKGGVRACSS
59	PBX1	7.3E-26	TGAKTGACAGS
60	KLF8	1.7E-24	CAGGGKGTG
61	ZIC1	7.0E-23	KGGGWGGTS
62	ERG	5.9E-22	VVRCMGGAAGYRVV
63	SRBP2	6.3E-20	VSGTGGGGWGABG
64	SRBP1	1.9E-18	GGTGGGGTGAB
65	USF1	3.7E-16	SRGTCACGTGRCS
66	MYCN	4.5E-13	SCACGTGG
67	E2F5	1.5E-12	SGCGCSAAAH
68	MXI1	1.8E-12	VCACGTGGSNGSBGS
69	MTF1	2.2E-12	MGKGCCGTGTGCAAARS
70	HINFP	4.0E-12	DMSHHMGCGGACGTTV
71	HEN1	8.8E-12	BGGGKMKCAGCTGCGKCCYY
72	INSM1	8.0E-11	TGTMAGGGGGCR
73	PAX5	6.8E-10	VDRVKCAGYSRAGCGTGAC
74	ZFP42	1.2E-09	GGCMGCCATTTT
75	ELF2	3.4E-09	TDNCAGGAAGTRRVT
76	OVOL2	9.4E-09	RTAACGGD
77	EPAS1	1.8E-07	VTACGTGMC
78	NR1H4	3.8E-07	RRGGKCAVTGRCCBSGVGG
79	HIF1A	4.2E-07	GDACGTGM
80	ZBT7A	3.4E-06	VRGGGKCKY
81	ZIC3	7.3E-06	GGCYHCCTGCTGWGH
82	ZN143	1.2E-05	KGCMTKCTGGGARWTGTAGTYY
83	GATA1	6.2E-05	TGSNSSNNDSAGATAAGRV
84	TFE3	1.1E-04	RGTCACGTGA
85	ZN322	9.1E-04	GAGCCTGSTACWSWGCCTGR
86	ZIC2	1.1E-03	DGCYHCCTGCTGWGH
87	COE1	3.4E-03	RGYCCCWGGGGAS
88	RFX3	3.6E-03	CSGTTGCCATGGMRAC
89	MITF	5.3E-03	YCWYGTGACY
90	TAL1	6.4E-03	CTGSNNNNNRSAGATAAGRV
91	PRDM5	1.1E-02	VMTGGAGADCMRGGW
92	EHF	1.2E-02	VWACSAGGAAGTDVS
93	ETV2	1.2E-02	RRARRCAGGAARYRGS
94	MYBA	1.8E-02	VGYGGCVGTTRSVGV

Supplemental Figure 5. **(A)** PCA plot of RNA-seq from sorted *Yy1*$^{+/+}$ and *Yy1*$^{-/-}$ Lin^{-} cKit^{+} E14.5 FL cells. **(B)** Heatmap depicting selected upregulated and down-regulated genes based on TPM. **(C)** PCA plot of CUT&Tag. **(D)** Duplication rate of each CUT&Tag sample. **(E)** Motif analysis identified 94 significantly enriched (adjusted P < 0.05) transcription factor motifs, ranked based on adjusted P. TF motifs known to play essential roles in hematopoiesis, chromatin structure regulation, cell stress response, and cellular proliferation are highlighted.

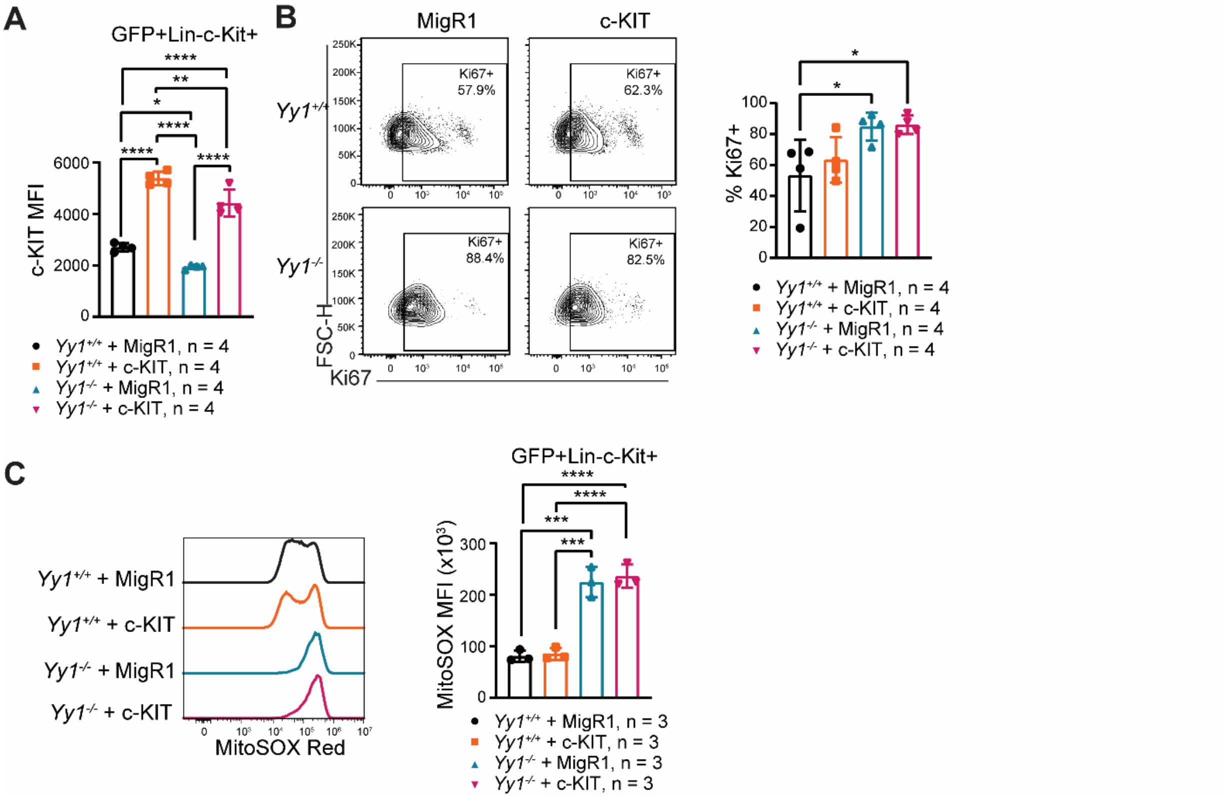

Supplemental Figure 6. Ectopic expression of c-Kit did not rescue YY1-dependent cell cycle or mitochondrial ROS in FL LK cells. (A) Ectopic expression of c-Kit rescued c-Kit MFI in YY1 deficient FL LK cells. **(B)** Representative gating strategy for Ki67 proliferation assay. E14.5 Lin$^-$ FL cells were infected with either MigR1 (control) or MigR1-c-Kit vectors. The percentages of Ki67$^+$ and Ki67$^-$ cells were quantified within GFP$^+$Lin$^-$c-Kit$^+$ populations. **(C)** Representative flow cytometry plots and quantification of mitochondrial ROS levels in GFP$^+$Lin$^-$c-Kit$^+$ cells infected with either MigR1-c-Kit or MigR1 vector control. N represents the number of fetuses; graphs show means ± SD, *P <0.05, **P <0.01, ***P<0.001, ****P<0.0001 by one way ANOVA.

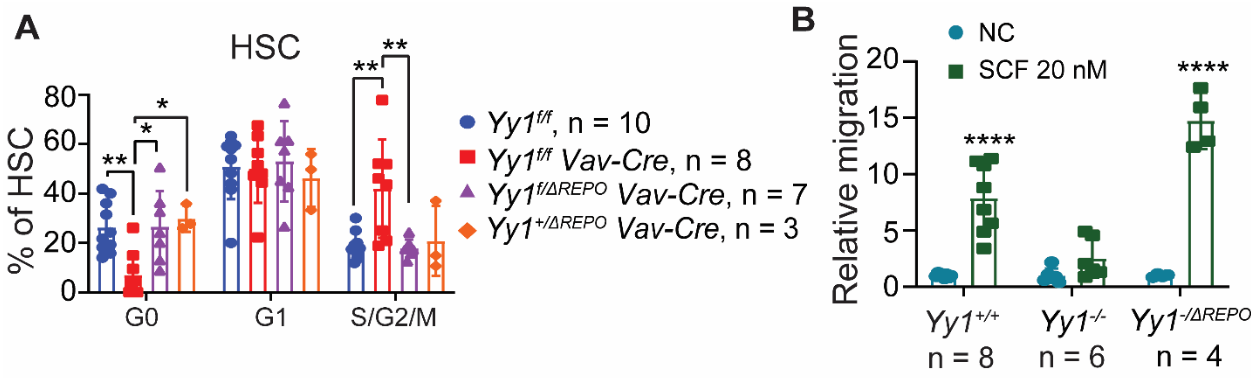

Supplemental Figure 7. The YY1 REPO domain deletion did not impact HSC proliferation and SCF-dependent cell migration. (A) Quantification of percentage of fetal HSC in G0 (Ki67$^-$DAPI$^-$), G1 (Ki67$^+$DAPI$^-$) and S/G2/M (Ki67$^+$DAPI$^+$) phases. (**B**) Ex vivo migration assay of *Yy1*$^{-/\Delta REPO}$, *Yy1*$^{-/-}$ *and Yy1*$^{+/+}$ Lin$^-$ FL cells in response to SCF stimulation. While YY1-deficient cells exhibit significantly impaired migration upon SCF stimulation, both wild type and *Yy1*$^{-/\Delta REPO}$ cells are responsive to SCF stimulation. N represents the number of fetuses; graphs show means ± SD, *P <0.05, **P <0.01, ****P<0.0001 by 2-way ANOVA.

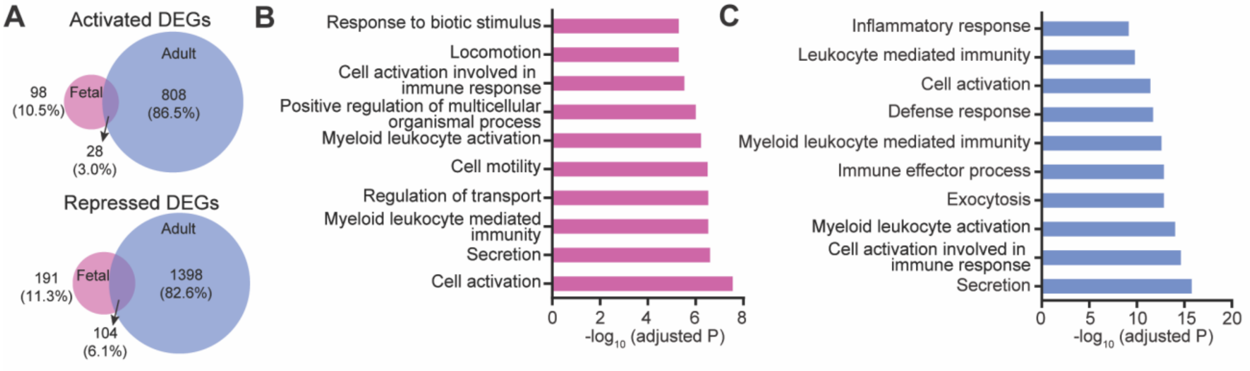

Supplemental Figure 8. (**A**) Venn diagram of activated and repressed DEGs associated with YY1 deletion in fetal versus adult HSPCs. (**B-C**) GSEA analysis of enriched GO pathways in fetal (**B**) and adult HSPCs (**C**).

Table 1

qRT-PCR Primer list

	Forward	Reverse
Yy1	TCAGACCCTAAGCAACTGGCAGAA	TTGAGCTCTCAACGAACGCTTTGC
Kit	AGTGGACGTACAGGTCCAGAA	GCCTGGATTTGCTCTTTGTTGT
S100a8	ATCACCATGCCCTCTACAAGAATG	GTCCAATTCTCTGAACAAGTTTTCG
S100a9	AAGCTGCATGAGAACAACCCA	CCCAGAACAAAGGCCATTGA
Gpc1	GAATTTGGCCAACCACAGCC	GTAGTAGAGGCGCAGCTCAG
Hck	GCCAACCTGATGAAGTCGCT	TGTACTCATTGTCCTCGATGATTCG
Csf1r	CAGTTCAGAGTGATGTGTGGTC	CTTGTTGTTCACTAGGATGCCG
Padi2	CAGCCGCCTATACGGGAAA	CTTCACACCTTCCGAGTGCT
Jam3	TACCGCAATGATGTGCCACT	GATGAAGCAGCCTCGTCTGT
Cd63	CTGTGGGCTGTGGGAATGAT	CCTCCACAAAAGCAATGCCC
Cxcr2	CATCCCGTTTGAGGGTCGTA	GCAGGAAGACAAGGACGACA
Mpo	CCTCCAACCGTGCCTTTGTA	GTCAGCTGATCGTTGGGGAA
Olr1	CTGCGAATGACGAGCCTGA	CCAGAGTCATAGCAGCAGGG
Pnck	ATCGCGGTACTTCGCAGAAT	CCAAGGACCTGCCCTACAAG
Cxcr4	GACTGGCATAGTCGGCAATG	AGAAGGGGAGTGTGATGACAAA
Gapdh	TCCTGCACCACCAACTGCTT	GTCTTCTGGGTGGCAGTGAT

Table 2

Genotyping Primer list

	Forward	Reverse
Yy1 P1, P2	ACCTGGTCTATCGAAAGGAAGCAC	GCTTCGCCTATTCCTCGCTCATAA
Yy1 P3, P4	TAGAGAATAGGAACTTCGGCCGCCA	CCAAAGTTCGAAACCTGCTTTCCT
$Yy1^{\Delta REPO}$	GACGGCTTCGAGGACCAGAT	GCGCCCATCACACACATAAAA
