# Supplementary material for: Chromatin factor YY1 controls fetal hematopoietic stem cell migration and engraftment in mice
Source: J Clin Invest. 2025 Jul 29;135(19):e188140. doi: 10.1172/JCI188140 (PMC12483607; doi:10.1172/JCI188140)
Supplement: Unedited blot and gel images [file jci-135-188140-s194.pdf]

Full unedited gel images for Figure 1C

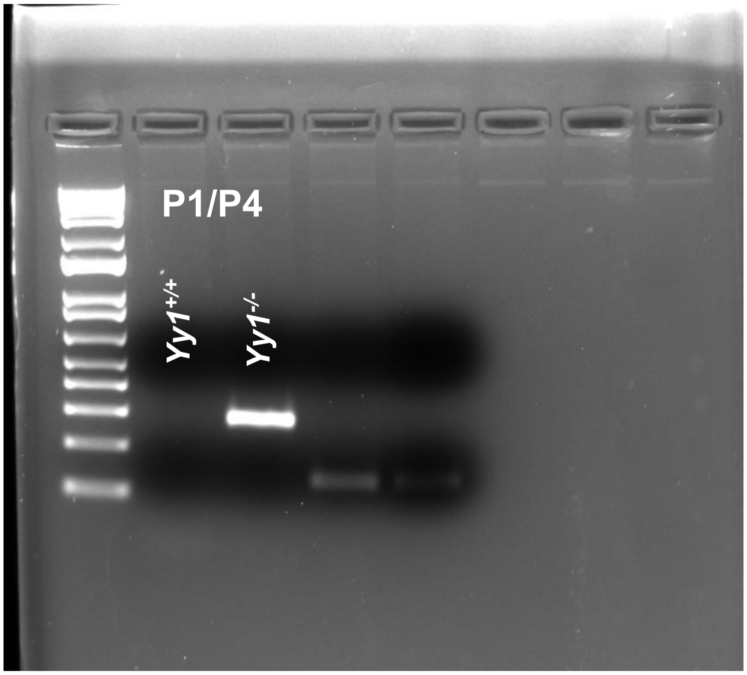

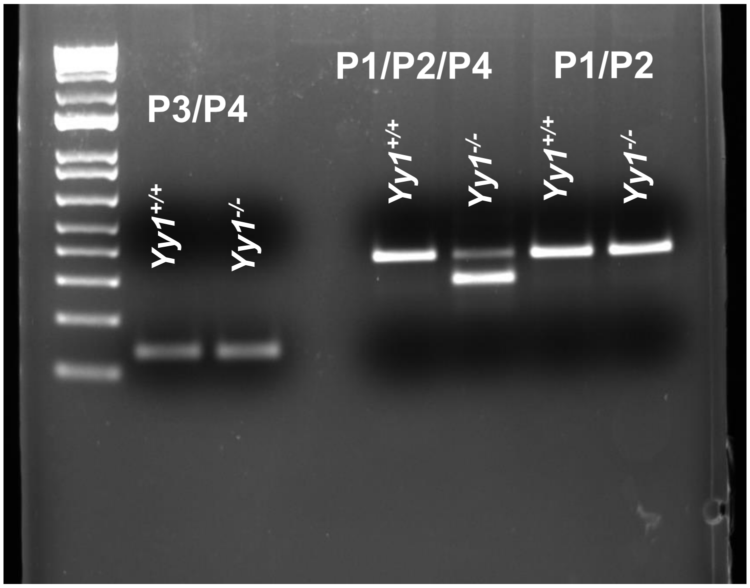

Full unedited gel image for Figure 8C

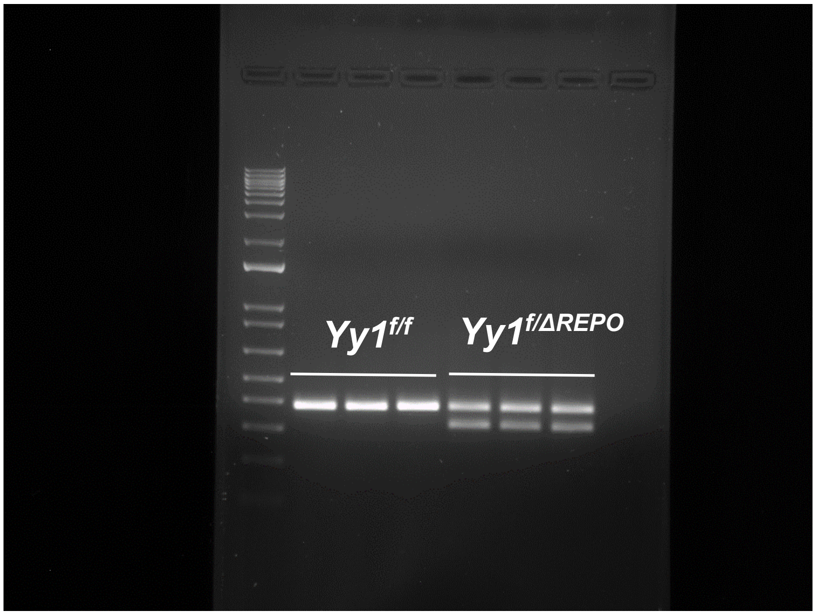

Full unedited blot image for Figure 8D

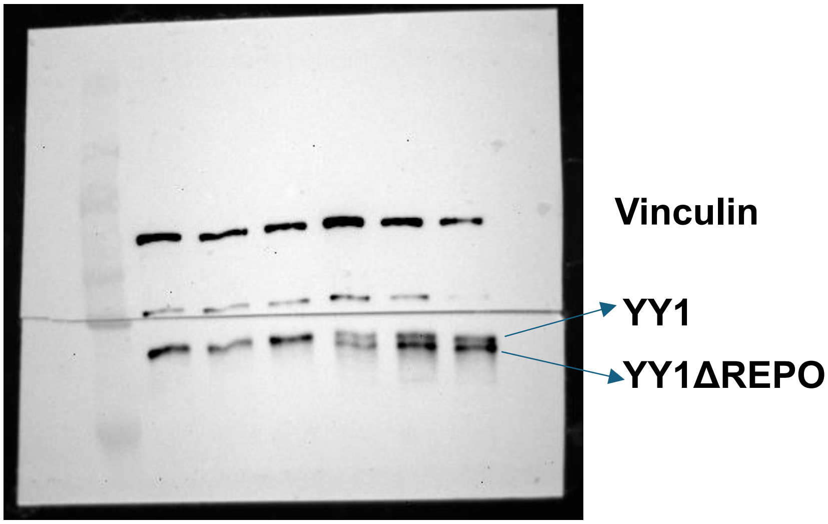

Full unedited blot images for Supplemental Figure 1C

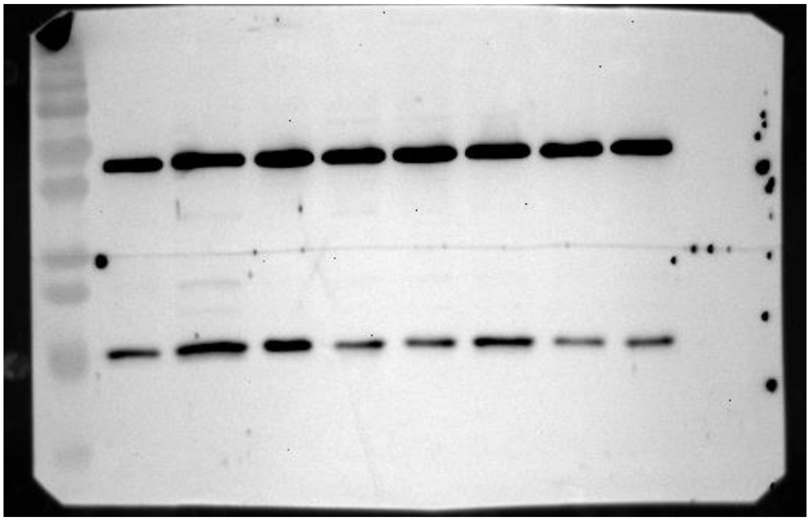

γH2AX

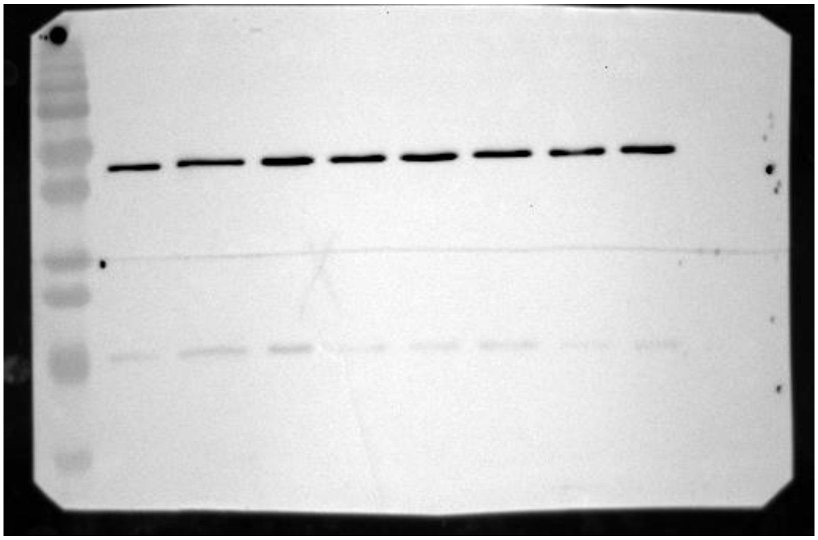

β-Actin
